# Bioinspired Macrocyclic Molecule Supported Two‐Dimensional Lamellar Membrane with Robust Interlayer Structure for High‐Efficiency Nanofiltration

**DOI:** 10.1002/advs.202206516

**Published:** 2022-12-21

**Authors:** Pengcheng Zhang, Yujuan Zhang, Lin Wang, Kaikai Qiu, Xiaoyi Tang, John K. Gibson, Xue Liu, Lei Mei, Shuwen An, Zhiwei Huang, Peng Ren, Yi Wang, Zhifang Chai, Weiqun Shi

**Affiliations:** ^1^ Laboratory of Nuclear Energy Chemistry Institute of High Energy Physics Chinese Academy of Sciences Beijing 100049 China; ^2^ Radiochemistry Laboratory School of Nuclear Science and Technology Lanzhou University Lanzhou 730000 China; ^3^ Engineering Laboratory of Advanced Energy Materials Ningbo Institute of Materials Technology&Engineering Chinese Academy of Sciences Ningbo 315201 China; ^4^ School of Materials Science and Engineering University of Science and Technology Beijing Beijing 100083 China; ^5^ Chemical Sciences Division Lawrence Berkeley National Laboratory Berkeley CA 94720 USA; ^6^ State Key Laboratory for Mechanical Behavior of Materials Xi'an Jiaotong University Xi'an 710049 China; ^7^ School of Nuclear Science and Engineering East China University of Technology Nanchang 330013 China; ^8^ State Key Laboratory of NBC Protection for Civilian Beijing 102205 China

**Keywords:** membrane separation, MXene, nanofiltration, size sieving, uranium enrichment

## Abstract

2D lamellar membranes (2DLMs) are used for efficient desalination and nanofiltration. However, weak interactions between adjacent stacked nanosheets result in susceptibility to swelling that limits practical applicability. Inspired by the super adhesion of multi‐point suction cups on octopus tentacles, a 2DLM is constructed from Ti_3_C_2_T*_x_* MXene supported by the macrocyclic “multi‐point” molecule cucurbit[5]uril (CB5) and demonstrated for nanofiltration of methyl blue (MB) and enrichment of uranyl carbonate. Experimental results and density functional theory calculations indicate that CB5 rivets to the surface of the nanoflakes through strong stable interactions between its multiple binding sites and surface hydroxyl functional groups on MXene nanosheets. This novel 2DLM exhibits excellent nanofiltration performance (69 L m^−2^h^−1^bar^−1^ permeance with 93.6% rejection for MB) and can be recycled at least 30 times without significant degradation. The 2DLM exhibits excellent swelling resistance at high salinity, with a demonstration of selective enrichment of uranyl carbonate from artificial water and natural seawater. The results provide a new strategy for constructing highly stable 2DLMs with interlayer spacing controllable from sub‐nano to nanometer scales, for size‐selective sieving of molecules and ions, high‐efficiency nanofiltration, and other applications.

## Graphical abstract

2D lamellar membranes (2DLMs) are promising for nanofiltration and desalination, but reported membrane materials suffer from swelling in aqueous solutions and structural instability under pressure. Inspired by the super adhesion of multi‐point suction cups on octopus tentacles, a 2DLM of MXene is constructed by intercalation of cucurbituril spacers, with resulting enhanced nanofiltration performance.

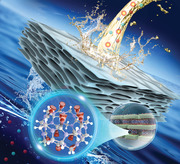

## 1 Introduction

Advanced separation technologies and materials are key for industrial applications such as water treatment, natural gas purification, and chemical separation. Among many available separation approaches for ions and molecules, membrane separation technology (MST) is generally preferred due to its advantages of cleanliness, ease of operation, and high efficiency.^[^ ^1^, ^2^, ^3^ ^]^ Compared with traditional organic/inorganic membrane materials, 2D materials featuring atomic‐scale thickness minimize transport resistance and offer new opportunities for MST.^[^ ^4^, ^5^, ^6^ ^]^ In general, separation membranes fabricated from 2D materials can be classified as either atomically thin nanoporous or laminar. Although the former is predicted to have excellent separation efficiency, in theory, the production of high‐quality large‐area porous monolayer nanosheets with controlled Ångstrom‐scale is extremely challenging.^[^ ^7^, ^8^ ^]^ Alternatively, 2D lamellar membranes (2DLMs) constructed by controlled stacking of nanosheets allow for precise manipulation of inter‐gallery channels, providing a convenient practical approach for high‐efficiency separations.^[^ ^9^, ^10^ ^]^ In the past decade, 2DLMs have aroused extensive interest due to their potential for ultra‐fast water transport behavior and selective sieving ability.^[^ ^2^, ^11^ ^]^ Interest in high‐performance 2DLMs has stimulated the exploration of new 2D materials, synthetic routes and manufacturing techniques, and studies to elucidate transport mechanisms in nanochannels.^[^ ^1^, ^4^, ^12^ ^]^

Because the transport of molecules and ions in 2DLMs occurs between adjacent nanosheets,^[^ ^13^, ^14^ ^]^ the interlayer spacing (d‐spacing) significantly affects selectivity and separation characteristics. Reliable control of this spacing is hindered by the weak nature of interactions between pristine nanosheets. Furthermore, oxygen‐containing surface functional groups prevalent in 2D materials may induce swelling of 2DLMs in polar solvents and lead to the expansion of d‐spacing.^[^ ^4^, ^15^ ^]^ A possible approach for improving the stability of 2DLMs while regulating d‐spacing is by incorporation of interlayer guests such as by immersing prepared 2D membranes in concentrated salt solutions to induce robust cross‐linking^[^ ^16^, ^17^ ^]^ or by intercalating large‐sized carbon nanotubes.^[^ ^18^, ^19^, ^20^ ^]^ However, preparation times for cross‐linking are long and result in non‐uniform ion distributions,^[^ ^16^ ^]^ while the latter is limited by the excessive occupation of interlayer channels, which slows down the water transport. Furthermore, these strategies have failed to achieve efficient stable separation efficiency for key target species with a dimension of around 1 nm. Selective retention of medium‐size molecules and large metal complex ions presents a clear need for new strategies to construct 2DLMs with suitable interlayer channels.

**Figure 1:**
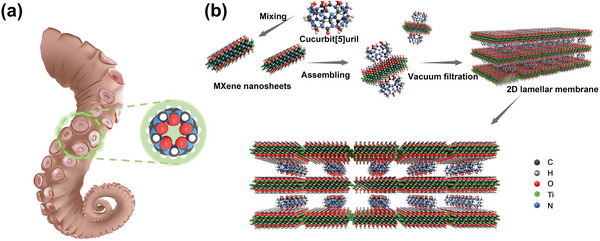
a) Biological inspiration and schematic of multiple CB5 binding sites. b) Schematic of fabrication procedure for CB5‐supported Ti_3_C_2_T*_x_* membrane.

As a relatively new type of 2D materials, MXenes (2D transition metal carbides, nitrides, and carbonitrides)^[^ ^21^, ^22^, ^23^, ^24^, ^25^ ^]^ have been used as water purification membranes, taking advantage of their negative surface charge, high hydrophilicity, and excellent mechanical properties.^[^ ^2^, ^4^, ^26^ ^]^ MXene‐based 2DLMs have shown superb abilities in the retention of proteins, antibiotics, and small‐sized hydrated metal ions from aqueous solutions.^[^ ^16^, ^27^, ^28^ ^]^ Opportunities remain for improved regulation of interlayer distances and membrane stability, particularly under conditions of high salinity and hydraulic pressure. Inspired by the super adhesion of multi‐point suction cups on octopus tentacles, we considered that multiple weak interaction sites between an intercalated guest and an MXene nanosheet should significantly enhance the overall structural stability of the lamellar membrane. As a well‐defined water‐soluble macrocyclic molecule,^[^ ^29^, ^30^ ^]^ cucurbit[5]uril (CB5) provides several binding sites at two symmetric portals, which might facilitate the assembly of MXene nanosheets. We here demonstrate this biomimetic strategy to stabilize the layered structure of Ti_3_C_2_T*_x_* membrane using CB5 (**Figure** **1**a), with the preparation of a high‐efficiency nanofiltration membrane. The CB5 units “rivet” to adjacent nanosheets to create stable spacers between them, resulting in a membrane that exhibits outstanding swelling resistance under high‐salt concentrations and hydraulic pressures, and expanded interlayer channels that facilitate water transport. Under conditions providing a rejection rate for methyl blue (MB) that is similar to alternative membrane materials, the water flux for the CB5‐stabilized MXene membrane is 2–3 times better. It is also demonstrated that the stable interlayer spacing results in the selective retention of uranyl carbonate from natural seawater.

## 2 Results and Discussion

### 2.1 Membrane Fabrication and Characterization

Figure 1b is a simplified representation of the procedure used to prepare the CB5‐supported MXene membrane. First, Ti_3_C_2_T*_x_* (TC) nanosheets uniformly dispersed in an aqueous solution (Figure S1, Supporting Information) were prepared using an in situ HF (LiF/HCl) etching and ultrasonic delamination method.^[^ ^31^ ^]^ Powder X‐ray diffraction (XRD) patterns (Figure S2, Supporting Information) confirmed the synthesis of TC nanosheets from Ti_3_AlC_2_ precursor by a shift of the (002) reflection and preferential orientation of (00*l*) basal reflections.^[^ ^15^, ^32^, ^33^ ^]^ Subsequently, CB5 was adsorbed onto the nanoflake surfaces through electrostatic interaction and the formation of hydrogen bonds between MXene surface hydroxyl groups and CB5 oxygen groups (Figure 1b). After spontaneous assembly using this procedure, the Ti_3_C_2_T*_x_*/CB5 membranes (TBM) were fabricated by traditional vacuum‐assisted filtration on polyethersulfone (PES) support (Figures S3 and S4, Supporting Information)^[^ ^34^, ^35^ ^]^ for characterization and nanofiltration tests (Figure S5, Supporting Information).

**Figure 2:**
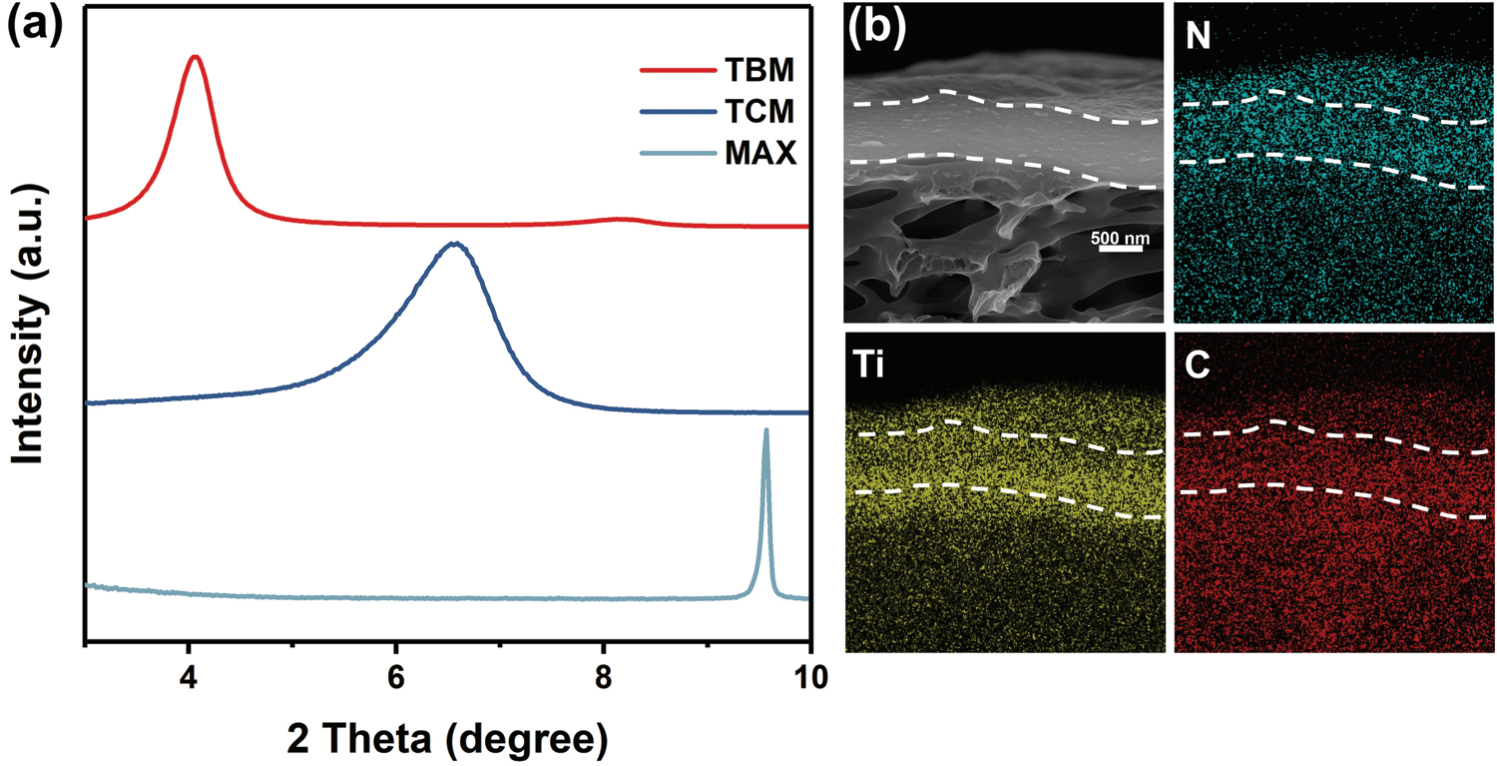
a) Comparison of XRD patterns of the precursor MAX phase, TCM, and TBM at low Bragg angles. b) Cross‐sectional SEM images of the TBM and elemental mapping images of N, Ti, and C from the same area.

**Figure 3:**
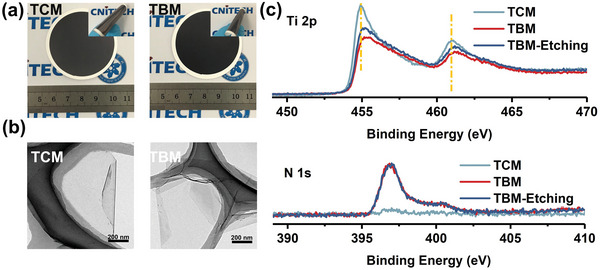
a) Optical photos of TCM and TBM. b) TEM images of nanosheets before and after loading with CB5. c) High‐resolution Ti 2p region and N 1s region of TCM, TBM, and TBM after etching for 180 s.

Small‐angle XRD patterns in **Figure** **2**a clearly show the expansion of the MXene interlayer spacing along the (002) plane induced by intercalation and assembly of CB5. The TBM (002) reflection at 2*θ* of ≈4.0° corresponds to an interlayer d‐spacing of ≈22 Å, which is ≈8.4 Å greater than for pristine Ti_3_C_2_T*_x_* membrane (TCM d‐spacing ≈13.6 Å based on 2*θ* ≈6.5°). This expansion in interlayer spacing is in reasonable correspondence to the predicted CB5 height of 9.1 Å (diameter 13.1 Å), indicating that CB5 assembles with Ti_3_C_2_T*_x_* nanosheets in an intercalation orientation with the portals of CB5 preferentially interacting with parallel MXene surfaces as shown in Figure 1b. The expansion of the interlayer spacing by a factor of ≈1.62 (i.e., 22 Å/13.6 Å) is reasonably consistent with variations in membrane thickness as a function of MXene loading, for which linear fits provide slopes for TBM and TCM with a ratio of ≈1.44 (Figures S6 and S7, Supporting Information). Thermogravimetry analysis of TBM provides a CB5 content of 20.8 wt% (Figure S8, Supporting Information). Energy‐dispersive X‐ray spectroscopy mappings in Figure 2b indicate that the pre‐assembly approach for preparing TBM results in CB5 is uniformly distributed among the TC nanoflakes. The optical image of 2DLMs and the morphology of MXene nanosheets are not altered significantly as a result of the addition of CB5 to yield TBM (**Figure** **3**a,b). The similar water contact angle of TCM and TBM, ≈53° and ≈61°, respectively (Figure S9, Supporting Information), indicate that the introduction of CB5 has a negligible effect on the hydrophilicity of the membrane surface.

X‐ray photoelectron spectra (XPS) of TCM, TBM, and TBM‐Etching (TBM after Ar^+^ sputtering) are shown in Figure 3c and Figure S10, Supporting Information. The N 1s signal is absent in TCM but appears in TBM with the intensity invariant after etching for 180 s (Figure 3c), corroborating that CB5 is homogeneously intercalated into the Ti_3_C_2_T*_x_* membrane. In the Ti 2p region,^[^ ^36^, ^37^ ^]^ a significant binding energy (BE) shift after intercalation can be observed as shown in Figure 3c. XPS peak fittings of Ti 2p spectra (Figure S11 and Table S1, Supporting Information) further reveal that the introduction of CB5 lead to a BE increase of 0.2, 0.2, and 0.1 eV for C—Ti—(O∖O∖O), C—Ti—(O∖O∖F), and C—Ti—(O∖F∖F) components, respectively, whereas the BEs of C—Ti—(F∖F∖F) and TiO_2‐_ *_x_* F_2_ *_x_* impurity remain unchanged. In addition, the fraction of Ti 2p fitted components in TBM is comparable with that in TBM‐Etching. The above analyses suggest that there is a uniform and strong interaction between CB5 and surface Ti atoms on MXene nanosheets in the TBM, in which oxygen‐containing groups may play an important role. Zeta potential measurements show that CB5 is positively charged in an aqueous solution (Figure S12, Supporting Information), which is the opposite of negatively charged Ti_3_C_2_T*_x_* MXene;^[^ ^38^ ^]^ resulting electrostatic attraction should provide a driving force for self‐assembly. The high affinity of CB5 for the MXene surface was further confirmed by UV–vis absorbance spectra titration (Figure S13, Supporting Information), which shows that colloidal Ti_3_C_2_T*_x_* nanosheets begin to agglomerate at very low CB5 concentration (≈25 mg L^−1^), suggesting that preparation of high‐quality TBM requires a very dilute solution to avoid agglomeration.

### 2.2 DFT Calculations of the Ti_3_C_2_T*_x_*‐CB5 Complex

**Figure 4:**
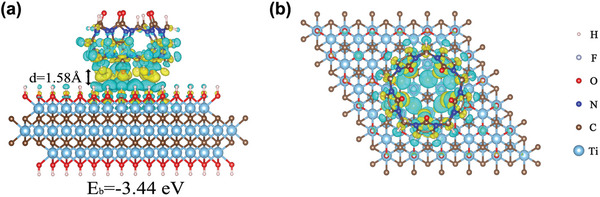
Side view (a) and top view (b) of the 3D electron density difference for CB5 adsorbed on Ti_3_C_2_(OH)_2_. The isosurface level is set to 0.001 e bohr^−3^. Yellow and blue colorings designate regions of charge accumulation and depletion, respectively.

Density functional theory (DFT) simulations were performed to provide insight into the interaction between CB5 and Ti_3_C_2_T*_x_* nanosheet, specifically Ti_3_C_2_(OH)_2_. The calculations reveal a distinctive electron transfer process from multiple adjacent hydroxyl groups on the nanosheet surface to carbonyl groups on the CB5 portal (**Figure** **4**), indicating enhanced host–guest binding due to synergism of multi‐site weak interactions. The binding energy (*E*_b_) of the Ti_3_C_2_(OH)_2_‐CB5 complex is calculated as −3.44 eV. The vertical distance between the nanosheet surface and the CB5 portal is computed as 1.58 Å, reflecting strong hydrogen bonding between MXene surface hydroxyls and cucurbituril carbonyls. It is well known that MXene surfaces are terminated by mixed —OH, —O, and —F functional groups as a result of the aqueous chemical etching process. For comparison with hydroxyl, computations were also performed for surface functional groups —O and —F (Figure S14, Supporting Information). The Ti_3_C_2_O_2_‐CB5 and Ti_3_C_2_F_2_‐CB5 surfaces exhibit less charge transfer than Ti_3_C_2_(OH)_2_‐CB5, perhaps due to the strong electronegativities of —O and —F terminations. For Ti_3_C_2_O_2_‐CB5 and Ti_3_C_2_F_2_‐CB5, the *E*_b_ values are −1.46 and −1.21 eV, and vertical separation distances are 2.23 and 2.01 Å. Compared with the hydroxylated surface, these binding energies are lower and distances longer, in accord with significantly weaker binding as indicated by the charge density distributions. The above calculation results reveal that the hydroxyl groups on the MXene surface are the most effective interaction sites for strong binding to CB5.

### 2.3 Swelling Resistance Evaluation

**Figure 5:**
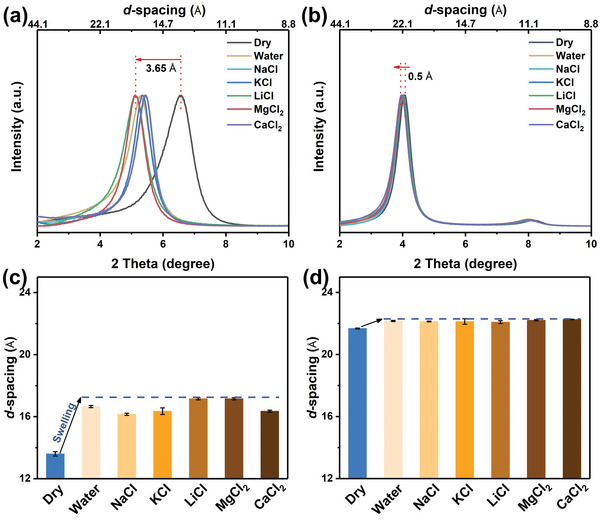
XRD patterns (a,b) and d‐spacings (c,d) for TCM and TBM in a dry state, and after immersion in DI water and various 0.2 m salt solutions for 4 h.

The structural stability of the as‐synthesized 2DLMs was assessed prior to nanofiltration testing since transport of water and solutes typically induces swelling of 2DLM structures,^[^ ^17^, ^39^, ^40^ ^]^ which can alter interlayer spacing and deteriorate sieving performance. As shown in **Figure** **5**, immersion of TCM in water, either pure or containing various metal ions, results in changes to XRD patterns that indicate swelling that increases the d‐spacing by ≈3.65 Å (Figure 5a,c). This result is similar to previous studies that show the intercalation of ions and water into TCM due to weak interlayer interactions.^[^ ^16^, ^41^ ^]^ In notable contrast to TCM, TBM exhibits only a slight (002) XRD peak shift (Figure 5b), corresponding to an increase in TBM d‐spacing of less than 0.50 Å (Figure 5d). Furthermore, the swelling behavior of TCM and TBM in different concentration gradients of salt was also studied (Figure S15, Supporting Information). The enlarged d‐spacing (Δd‐spacing) of TCM has a decrease from 3.12 to 2.56 Å with the increase of NaCl concentration, again confirming the flexible swelling nature of TC; while TBM shows a nearly constant Δd‐spacing (≈0.47 Å), suggesting a satisfactory swelling resistance either in dilute or in concentrated salt solutions. This excellent anti‐swelling behavior of TBM presumably reflects the larger interlayer spacing that more readily accommodates ions, and also the higher stability of the layer structure due to the “rivet” effect of CB5 between nanosheets. The demonstrated excellent swelling resistance suggests TBM as a good candidate for separation and filtration under saline conditions.

### 2.4 Nanofiltration Performance and Mechanism

**Figure 6:**
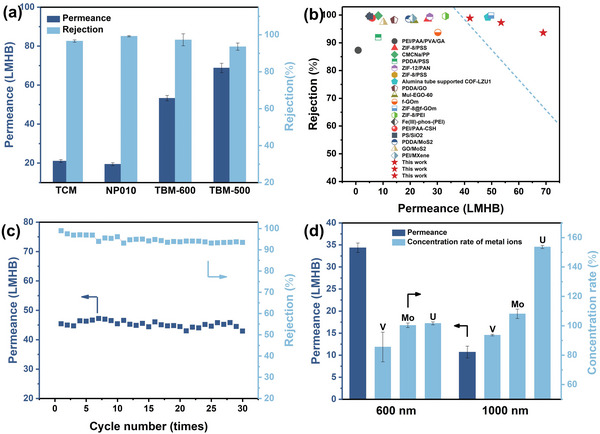
a) Nanofiltration performance of TCM, NP010, and TBM (500 and 600 nm thickness) for MB molecules at room temperature. b) Comparison of performance of TBM and other reported membranes. c) Performance of TBM (thickness 700 nm) after multiple use cycles. d) Separation of V/Mo/U in natural seawater by TBM with 600 and 1000 nm thickness.

Considering the channel size established above by the TBM interlayer distance of ≈8–9 Å, the bulky highly‐charged dye molecule MB (dimensions ≈23.6 Å × 17.4 Å × 8.0 Å, Figure S16, Supporting Information) was employed to evaluate nanofiltration performance. Initial tests of performance versus the amount of added CB5 (*V*_CB5_) revealed a trade‐off between water permeance and efficiency rejection of MB (Figure S17a, Supporting Information).^[^ ^42^ ^]^ Upon initial addition of CB5 to generate TBM, the interlayer spacing increases gradually (Figure S17b, Supporting Information), thus leading to an increase in water flux and a decrease in rejection rate; additional CB5 after intercalation saturation results in significantly decreased permeance accompanied by a slight increase in MB rejection. These results suggest that additional CB5 beyond saturation does not increase the interlayer spacing but rather blocks membrane channels and increases flow resistance. Based on the trade‐off between simultaneously optimizing permeance and MB rejection, a *V*_CB5_ of ≈116 µL per 1.55 mg MXene was used for the performance assessment described below. Another key performance parameter is the thickness of the TBM membrane. Results for MB rejection in Figure S18a, Supporting Information, suggest a thickness in the range of 500–700 nm is found to exhibit a promising trade‐off between flux (42–69 L m^−2^h^−1^bar^−1^, dimensions abbreviated as LMHB) and retention of MB (99.1–93.6%). The invariance of the XRD pattern shows that the membrane thickness has a negligible effect on interlayer spacing (Figure S18b, Supporting Information), indicating excellent structural stability of TBM. Comparison of nanofiltration performance of two TBM thicknesses with a commercial PES membrane (NP010) and TCM under the same conditions, shown in **Figure** **6**a, reveals the permeance for the TBM is ≈2.5–3.5 times higher than for the other two, with comparable MB retention for all four. Better flow through TBM versus TCM clearly reflects the increased interlayer spacing due to CB5 spacers in TBM. A comparison of the MB nanofiltration performance of TBM with several other materials reported in the literature, such as polyelectrolyte membranes, metal‐organic framework composite membranes, covalent organic framework composite membranes, and common 2DLMs of MoS_2_, GO, and MXene, is summarized in Figure 6b and Table S2, Supporting Information. All the materials exhibit comparable MB rejection rates, whereas TBM exhibits a permeance significantly higher than most state‐of‐the‐art membranes, which are mostly in the range of 10–30 LMHB, demonstrating the advantages of CB5‐Ti_3_C_2_T*_x_* assembly strategy for producing high‐performance 2DLMs.

For application in large‐scale separations and filtration, a membrane should exhibit high‐efficiency performance, durability, and pressure resistance under typical operating conditions.^[^ ^43^ ^]^ Considering that the permeate continuously accumulates under dead‐end filtration conditions, a multiple‐cycle approach is used to evaluate durability. As shown by the results in Figure 6c, TBM maintains performance for at least 30 filtration cycles, demonstrating excellent reusability. The stability of the membrane structure is corroborated by XRD after cycling (Figure S19, Supporting Information), which shows a negligible shift in the (002) peak and no impurity phases. The pressure dependence of TBM separation performance is assessed by the results in Figure S20a, Supporting Information. Increasing the applied pressure from 1 to 5 bar results in a nearly linear increase in water flux to ≈160 Lm^−2^h^−1^ (LMH), with the rejection rate remaining above 90% up to 3 bar, and decreasing to ≈80% at 5 bar. When the feed pressure was restored to 1 bar, the membrane performance was almost as good as before pressure testing and the interlayer spacing was essentially unchanged (Figure S20b, Supporting Information). These results reflect that the highly stable TBM is capable of high‐efficiency retention of MB under moderate pressure. Based on the above evaluation, it is apparent that the TBM exhibits excellent nanofiltration performance under actual application conditions.

To clarify the separation process, UV–vis absorption spectra of the retentate were obtained after different operating times.^[^ ^44^, ^45^ ^]^ The MB concentration on the feed side increased continuously with time (Figure S21a, Supporting Information), which corresponds to the concentration effect induced by size rejection during membrane separation. Furthermore, we measured the spectra and determined the feed concentration of the dye solution, as well as the concentration of the retentate and permeate after membrane separation (Figure S21b, Supporting Information). It is found that the total amount of dye molecules in the permeate (0.2 mg L^−1^, 17.1 mL) and retentate (13.5 mg L^−1^, 42.8 mL) sides are very close to that in the original feed (10 mg L^−1^, 60 mL). Additionally, in a 4‐h static soaking experiment, the UV–vis curves before and after adsorption were essentially invariant (Figure S21c, Supporting Information), suggesting very low adsorption of MB by TBM. These results suggest that a primary interception mechanism is size repulsion, rather than physical or chemical adsorption by TBM. In addition to size sieving, surface charge interaction is also involved in membrane separation. Zeta potentials of MXene, MXene‐CB5 nanosheets, and MXene‐CB5 agglomerates (Figure S22, Supporting Information) at different solution pHs clearly confirm that the surface charges of all three samples are negative under near‐neutral pH conditions, although the value of MXene‐CB5 agglomerates is close to zero due to the excessive introduction of CB5. Under the same addition amount of CB5 as that of TBM fabrication, the prepared MXene‐CB5 nanosheets show zeta potentials around −40 mV at pH ≈6–8, implying that the surface and transport channels of TBM have strong electrostatic repulsion against negatively charged MB molecules. Therefore, it can be concluded that the efficient separation of MB by TBM is ascribed to the synergistic effect of size rejection and surface charge repulsion.

### 2.5 Enrichment of Uranium from Artificial Water and Natural Seawater

Extraction of uranium from seawater is attractive for the development of nuclear energy since the ≈4.5 billion tons of dissolved uranium is ≈1000 times the terrestrial inventory.^[^ ^46^, ^47^ ^]^ However, separation from seawater is hindered by low uranium concentration, high salinity, and interference from other metals. Seawater uranium is mainly in the form of uranyl carbonate anion, [UO_2_(CO_3_)_3_]^4−^, which has an effective diameter of ≈9.7 Å in the equatorial plane that renders it among the largest marine metal ions.^[^ ^48^ ^]^ Because most hydrated metal ions exhibit an effective diameter of less than 9 Å,^[^ ^49^ ^]^ the MST based on size and charge repulsion presents an attractive strategy for uranium extraction.

Given the good performance of TBM using dye molecule MB, this membrane material was evaluated for separating uranyl carbonate from artificial water and natural seawater. Using an aqueous solution of uranyl carbonate with a uranium concentration of 10 mg L^−1^, the retention efficiency of the 600 nm thick membrane (TBM‐600) exceeded 97.5% with a water permeance of ≈45 LMHB (Figure S23a, Supporting Information). Despite good performance with the aqueous solution, for simulated seawater spiked with uranium (Figure S24a, Supporting Information) only 18% of uranyl carbonate was retained by the 600 nm thick TBM. This dramatic decrease in retention efficiency is attributed to two degrading effects: decreased Debye screening length; and non‐uniform distortion of membrane interlayer spacing. The Debye screening length of an electrolyte solution is inversely proportional to the square root of concentration.^[^ ^50^, ^51^ ^]^ According to the Donnan exclusion theory,^[^ ^52^ ^]^ at high salt concentration, electrostatic repulsion due to intrinsic charge on a membrane surface will decrease in parallel with decreasing Debye screening length, resulting in more facile transport of negative ions like uranyl carbonate. The second degrading effect, membrane distortion, is revealed by the XRD patterns of TBM after the single‐component and artificial seawater tests (Figures S23b and S24b, Supporting Information). Broadening of the (002) reflection after exposure to the highly saline artificial seawater reveals a non‐uniform distribution of the interlayer spacing that may lead to a decrease in the rejection rate of uranyl carbonate. The above results and analyses also reveal that both the charge and size effect are responsible for the retention of uranyl carbonate complex ions. With the goal of improving the structural stability of the membrane and lengthening the diffusion pathway, a TBM with a thickness of 1000 nm (TBM‐1000) was prepared and tested (Figure S23a and S24a, Supporting Information). As expected, this TBM‐1000 membrane showed an improved rejection rate for uranyl of 47% while maintaining low rejection rates of ≈5% for Na^+^, K^+^, Ca^2+^, Mg^2+^, and Sr^2+^, establishing selective enrichment of uranium by TBM under high salinity conditions, albeit with reduced permeance with increased membrane thickness.

To further evaluate the practical separation performance of TBM, unspiked natural seawater was used as the feed solution. The concentrations of uranium, molybdenum, and vanadium were determined before and after the nanofiltration, with the latter two metals selected because they are in seawater at concentrations similar to uranium, and also as anionic complexes, MoO_4_^−^ and VO_2_(OH)_2_^−^/VO_3_ (OH)^−^. As shown in Figure 6d, TBM‐600 exhibits negligible enrichment for the three metal ions, presumably due to surface charge screening and the broadening of interlayer spacing under high salinity conditions. As for TBM‐1000, the concentration rate of uranium exceeds 150%, while vanadium and molybdenum are not significantly enriched, a result that indicates the successful separation of uranium from seawater. XRD patterns (Figure S25, Supporting Information) obtained before and after testing with nature seawater demonstrate better structural stability and salinity resistance of the more uranyl‐selective TBM‐1000 versus TBM‐600. These results are consistent with the results of the simulated seawater test.

## 3 Conclusion

In summary, Ti_3_C_2_T*_x_* MXene and CB5 were employed as rigid building blocks to assemble a novel 2D lamellar membrane TBM with expanded interlayer channels and excellent nanofiltration performance. Experimental and theoretical results indicate strong binding between Ti_3_C_2_T*_x_* nanosheets and CB5 through multi‐point interactions resulting in the ultra‐stable layered structure of TBM. This bioinspired membrane exhibits a remarkable retention effect on the model dye molecule methyl blue, with a water flux superior to previously reported membranes. Benefitting from good swelling resistance at high salinity, TBM exhibits partial separation of uranyl carbonate from simulated seawater, while prevalent metal ions like Na^+^ and Mg^2+^ are efficiently transported. Results for natural seawater confirmed selective uranium enrichment by TBM. The underlying retention mechanism was elucidated as synergistic effects of size sieving and charge repulsion. This work presents a new strategy for the construction of 2DLMs based on the control of stable nanosized interlayer spacing, with potential applications including nanofiltration of dye molecules and selective enrichment of large and multi‐charged metal ions.

## 4 Experimental Section

Methods and associated references are available in the Supporting Information.

## Conflict of Interest

The authors declare no conflict of interest.

## Supporting information

Supporting Information

## Data Availability

Research data are not shared.
