# Supplementary material for: Bioinspired Macrocyclic Molecule Supported Two‐Dimensional Lamellar Membrane with Robust Interlayer Structure for High‐Efficiency Nanofiltration
Source: Adv Sci (Weinh). 2022 Dec 21;10(5):2206516. doi: 10.1002/advs.202206516 (PMC9929118; doi:10.1002/advs.202206516)
Supplement: Supplementary file 1 — Supporting Information [file ADVS-10-2206516-s001.pdf]

ADVANCED SCIENCE
Open Access

Supporting Information

for *Adv. Sci.*, DOI 10.1002/advs.202206516

Bioinspired Macrocyclic Molecule Supported Two-Dimensional Lamellar Membrane with Robust Interlayer Structure for High-Efficiency Nanofiltration

Pengcheng Zhang, Yujuan Zhang, Lin Wang, Kaikai Qiu, Xiaoyi Tang, John K. Gibson, Xue Liu, Lei Mei, Shuwen An, Zhiwei Huang, Peng Ren, Yi Wang, Zhifang Chai and Weiqun Shi**

Supporting Information

Bio-inspired macrocyclic molecule supported two-dimensional lamellar membrane with robust interlayer structure for high-efficiency nanofiltration

Pengcheng Zhang[#], Yujuan Zhang[#], Lin Wang, Kaikai Qiu, Xiaoyi Tang, John K. Gibson, Xue Liu, Lei Mei, Shuwen An, Zhiwei Huang, Peng Ren, Yi Wang, Zhifang Chai, Weiqun Shi**

[#]These authors contributed equally to this work.

*Corresponding authors. E-mail: linwang@ihep.ac.cn; shiwq@ihep.ac.cn

1. Experimental Section

1.1. Preparation of $Ti_3C_2T_x$ Nanosheets

Ti_3AlC_2 powders (400 mesh) were purchased from Jilin 11 Technology Co., Ltd. A total of 1.0 g of Ti_3AlC_2 was slowly added to 10 mL of solution containing 9 M HCl and 1.0 g of LiF (Aladdin). The mixture was stirred at 35 °C for 24 hours, after which the product was centrifuged at 3500 rpm and washed 6 times with deionized water untill the pH reached ~6. $Ti_3C_2T_x$ nanosheets solution was produced from the exfoliation process of resultant slurry under ultrasonication and centrifugation. Ultrasonication was for 1 h, and centrifugation was for 60 min at 3500 rpm. The resulting supernatant was kept under inert gas. The $Ti_3C_2T_x$ concentration was calculated from the weight of dry $Ti_3C_2T_x$ nanosheets.

1.2. Preparation of Cucurbit[5]uril solution

Cucurbit[5]uril (CB5) was prepared according to the procedures reported previously.[1, 2] The obtained solid powder was further purified with 3M hydrochloric acid to remove a small amount of Cucurbit[7]uril. A certain mass of purified solid CB5 powder was weighed and dissolved in deionized water to prepare a transparent solution with concentration 5 mg mL^{-1}.

1.3. Preparation of $Ti_3C_2T_x$-Cucurbit[5]uril mixture solution

Take a certain volume of Cucurbit[5]uril solution, disperse in 100 mL of deionized water, and continue to stir until the dispersion is uniform. Subsequently, the $Ti_3C_2T_x$ nanosheets were slowly added, after the solution is uniformly dispersed into dark green, the membrane can be prepared. Typically, 0.232 mL of Cucurbit[5]uril solution was disperse in 155 mL deionized water, and the amount of $Ti_3C_2T_x$ nanosheets was controlled at 3.09 mg (the loading amount here is defined as 3.09 mg).

1.4. Preparation of $Ti_3C_2T_x$ and $Ti_3C_2T_x$-Cucurbit[5]uril membrane (TCM/TBM)

$Ti_3C_2T_x$ and $Ti_3C_2T_x$-Cucurbit[5]uril membrane were prepared via the vacuum filtration (**Figure S3**) of the corresponding solution on polyethersulfone (PES) support(220 nm, 50 mm, purchased from Jinteng) (**Figure S4**). The loading amount (thicknesses) of the membranes were tunable via adjusting the volume of the filtered solutions.

1.5. Materials Characterization

The X-ray diffraction (XRD) patterns were performed using a Bruker D8 Advance with filtered Cu-Kα radiation. The structure and morphology were characterized by scanning electron microscopy (SEM, Hitachi S-4800) and transmission electron microscope (TEM, JEOL 2100F). X-ray photoelectron spectroscopy (XPS) analysis was performed using an Axis Ultra DLD (Kratos) with monochromated Al-Kα radiation (1486.69 eV) under a pressure of 2×10^{-9} Tor. The ultraviolet-visible (UV-vis) absorbance spectra were collected from Hitachi UH-4150 spectroscopy. A Q500 thermogravimetry instrument (TA Instruments) was used to acquire thermogravimetric curves over the range of 20-800°C with a heating rate of 5 °C min^{-1} in air. The concentrations of uranium and other metal elements were determined by an inductively coupled plasma optical emission spectrograph (ICP-OES, Horiba JY2000-2, Japan) and inductively coupled plasma mass spectrometry (ICP-MS, Thermo Electron Corp. Thermo X7, USA).

1.6. Evaluation of separation performance

The Methyl Blue (MB) was purchased from Innochem (Beijing) Technology Co., Ltd. The natural seawater was collected from coastal water near the Zhoushan Islands (Zhejiang Province, China). MB and uranyl solution were filtrated through the prepared membranes with effective area of about 12.69 cm^2. The pressure-dependent filtration experiment is performed on a commercial bench-scale stainless steel dead-end stirred cell-filtration unit (Sterlitech HP4750, **Figure S5**). In general, 250 ml of the feed solution containing a certain solute (10 ppm, MB) was filtered across the membrane. The permeation time is about 1 h. The concentration of the molecules/ions in the permeate and retentate solutions were detected by ultraviolet–visible spectroscopy (Hitachi UH-4150)/ICP-OES/ICP-MS. The water flux was calculated based on the amount of the collected permeate after a certain separation duration. All the data of rejection rates and water fluxes were obtained from the average value of the measurements of three individual membranes. Except for the pressure dependence test, other

permeation tests were measured with pressure difference of 1 bar. The water flux J was calculated as the following equation:

$$J = V/St$$

where S is the effective area of the membrane, and t is the operation time.

The rejection was calculated as the following equation:

$$R\% = \frac{C_0 - C}{C_0}\%$$

where R is the rejection efficiency, C_0 and C are the molecule concentrations in the original feed side and the permeate side solutions, respectively.

1.7 DFT calculation Method

First-principles calculations were done by using the Vienna Ab-initio simulation package known as the VASP code. The electronic-ion interaction is characterized by the projector augmented wave method (PAW). The energy cut off of the plane waves was set to 450 eV. The electronic exchange-correlation function was treated using a generalized gradient approximation (GGA) in the form proposed by Perdew, Burke, and Ernzerhof (PBE). Both atomic positions and lattice vectors were fully optimized using the conjugate gradient (CG) algorithm with an energy precision of 10−5eV until the maximum atomic forces are smaller than 0.05 eV $Å^{-1}$. A vacuum region of 30 Å was adapted to eliminate the interaction of two adjacent slabs along z-axis. The Brillouin zone (BZ) integration was sampled by using a 2×2×1 Monkhost-Pack k-point grid centered on the gamma point for the 5×5×1 supercell. Grimme's DFT-D3 method was used to correct van der Waals forces between materials.

We detected the charge transfer of each part of CB5-$Ti_3C_2T_x$ complex. The difference in charge density distribution can be evaluated as the charge density distribution of CB5-$Ti_3C_2T_x$ complex ($\rho_{\text{CB5-Ti3C2Tx}}$) minus the charge density distribution of both parts of CB5 and $Ti_3C_2T_x$ nanosheet (ρ_{CB5} and ρ_{Ti3C2Tx}). No matter what kind of surface termination groups, we can intuitively observe the charge transfer between the two parts through multiple interactive sites.

The binding energy can be obtained as follows:

$$E_{\text{b}} = E_{\text{CB5-Ti3C2Tx}} - E_{\text{CB5}} - E_{\text{Ti3C2Tx}}$$

where $E_{\text{CB5-Ti3C2Tx}}$ is the total energy of CB5-$Ti_3C_2T_x$ (T_x = −OH, −O and −F) complex; E_{CB5} and E_{Ti3C2Tx} are the total energies of CB5 molecule and $Ti_3C_2T_x$, respectively.

2.Supplementary Figures

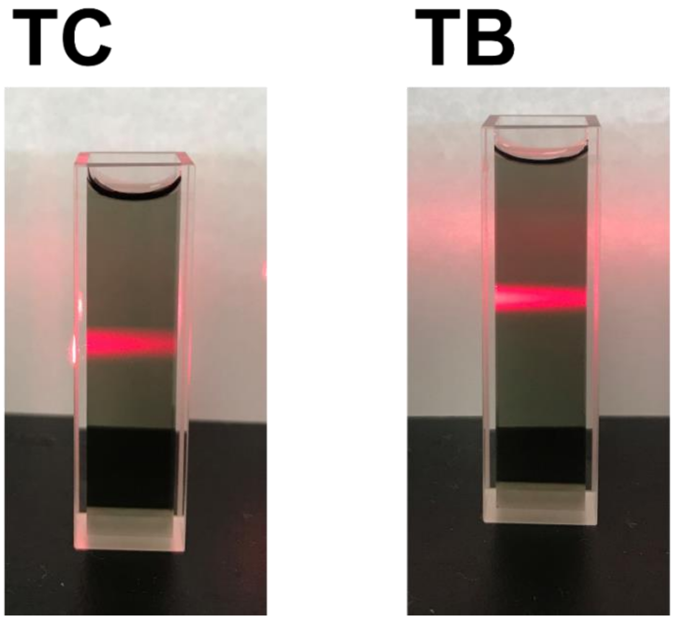

Figure S1. The Tyndall effect of the colloidal solution of TC and TB, after adding CB5, no obvious coagulation phenomenon was observed, and the dispersion of nanosheets was basically the same as that of TC.

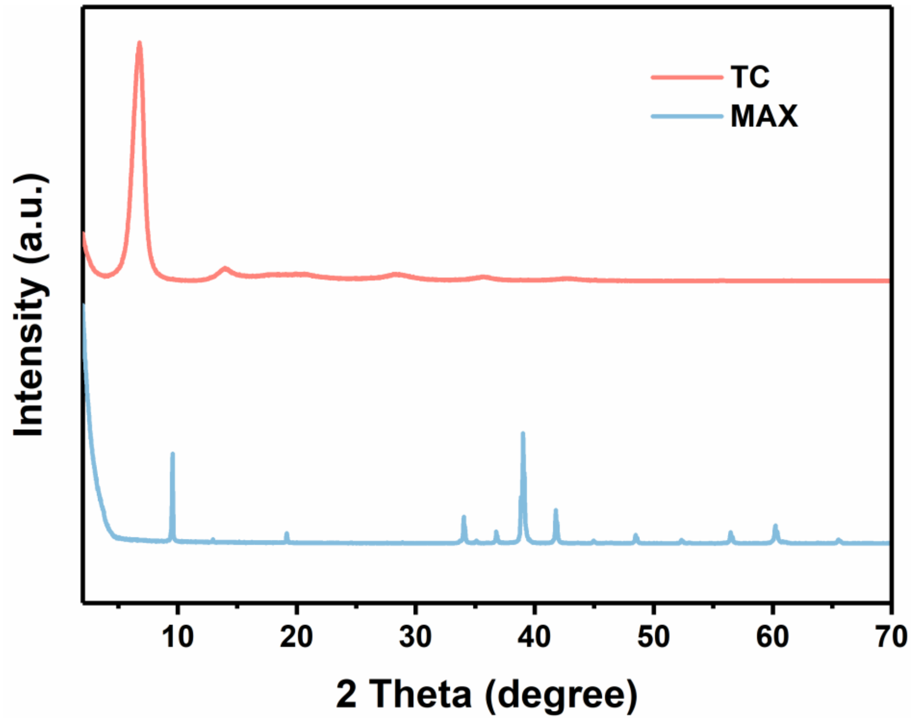

Figure S2. The XRD pattern of TC and MAX phase. The disappearance of the peak at 39 degrees proves that Al was successfully etched away, and disappearance of that at 61 degrees reflects the successful fabrication of the nanosheets.

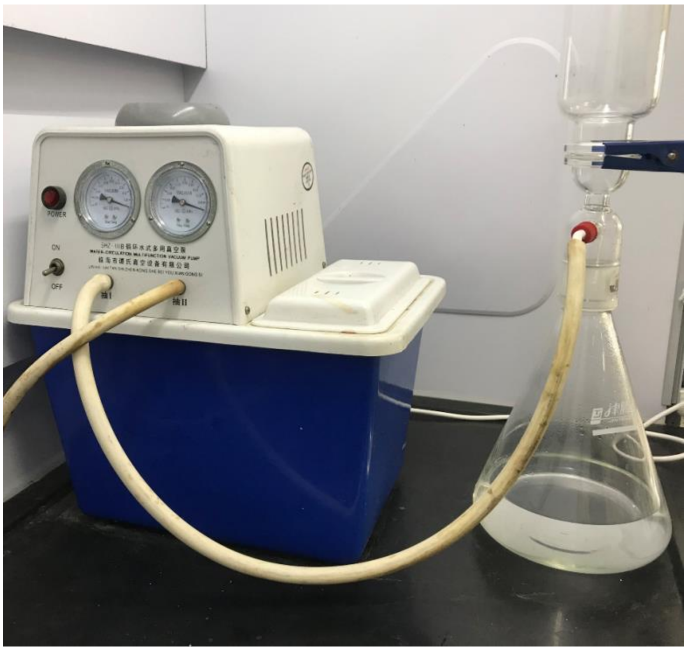

Figure S3. Device for membrane fabrication by vacuum filtration.

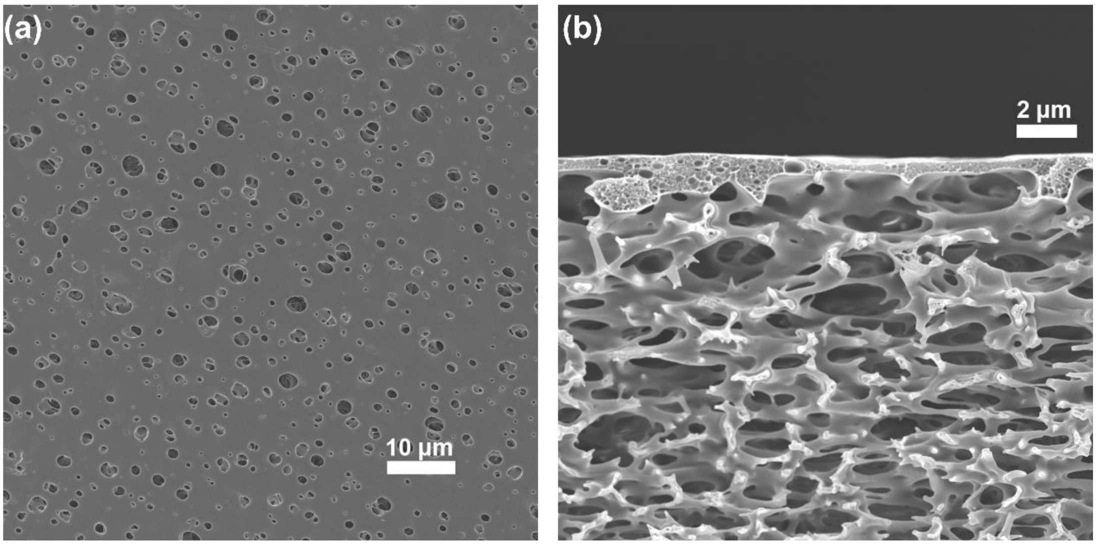

Figure S4. (a) Top view and (b) cross-sectional view of SEM images of porous PES support.

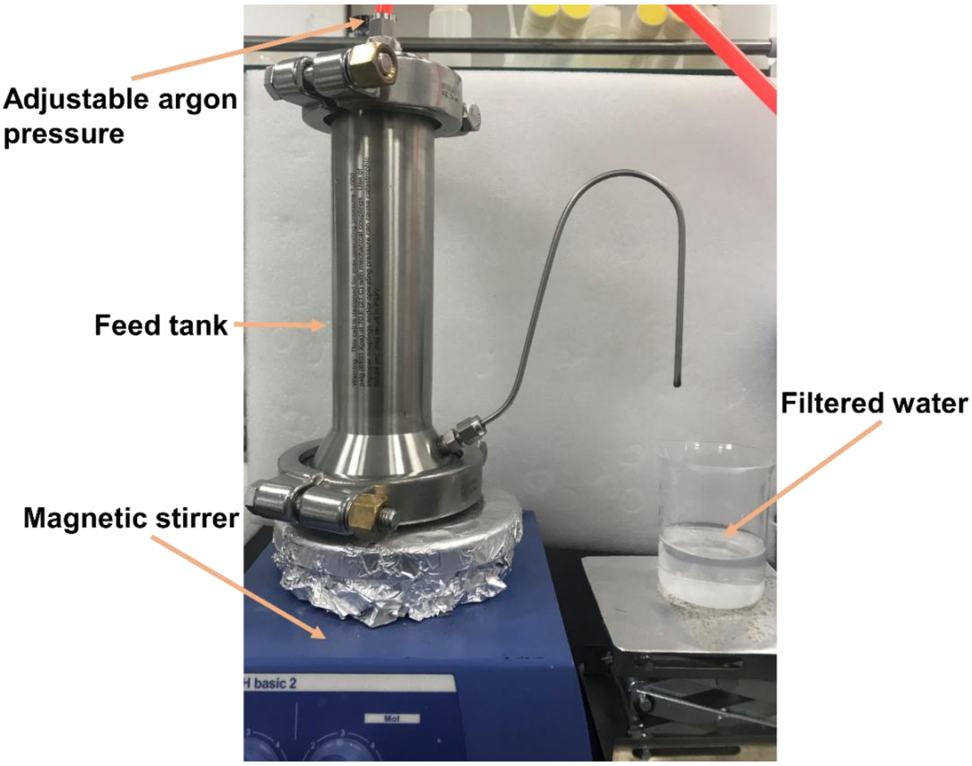

Figure S5. A dead-end filtration cell (Sterlitech HP4750 with high pressure stirred cell) pressurized by argon gas was used to examine the performance of the membranes.

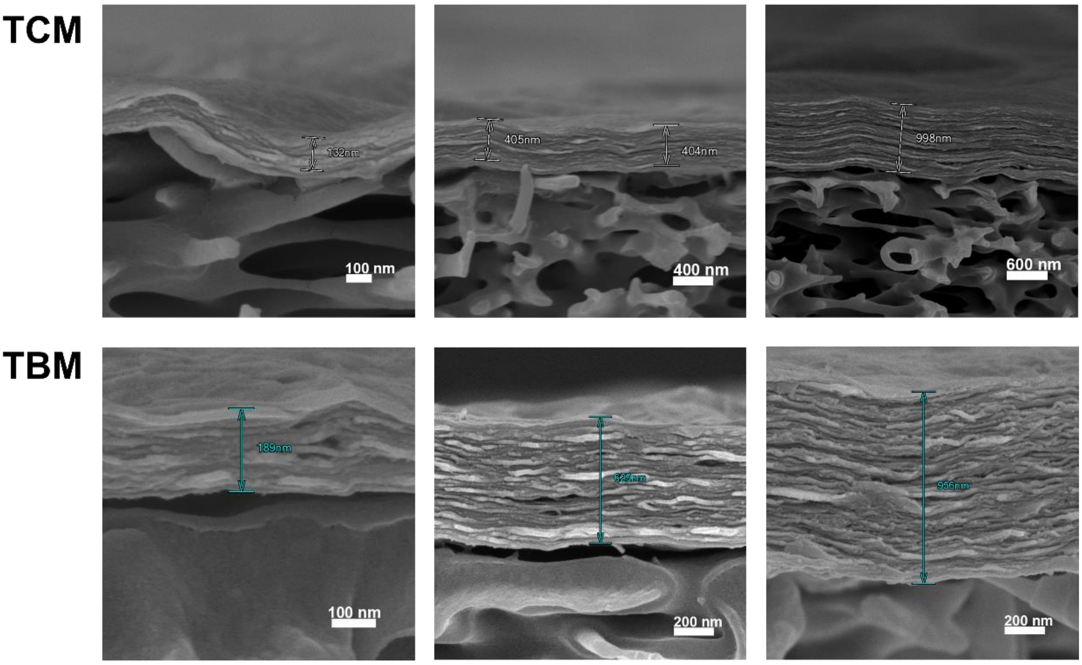

Figure S6. Cross-sectional SEM images of the TCM and TBM. The loading amount of MXene from left-to-right are: 0.62 mg, 1.86 mg and 4.86 mg for TCM; 0.78 mg, 1.95 mg and 2.93 mg for TBM.

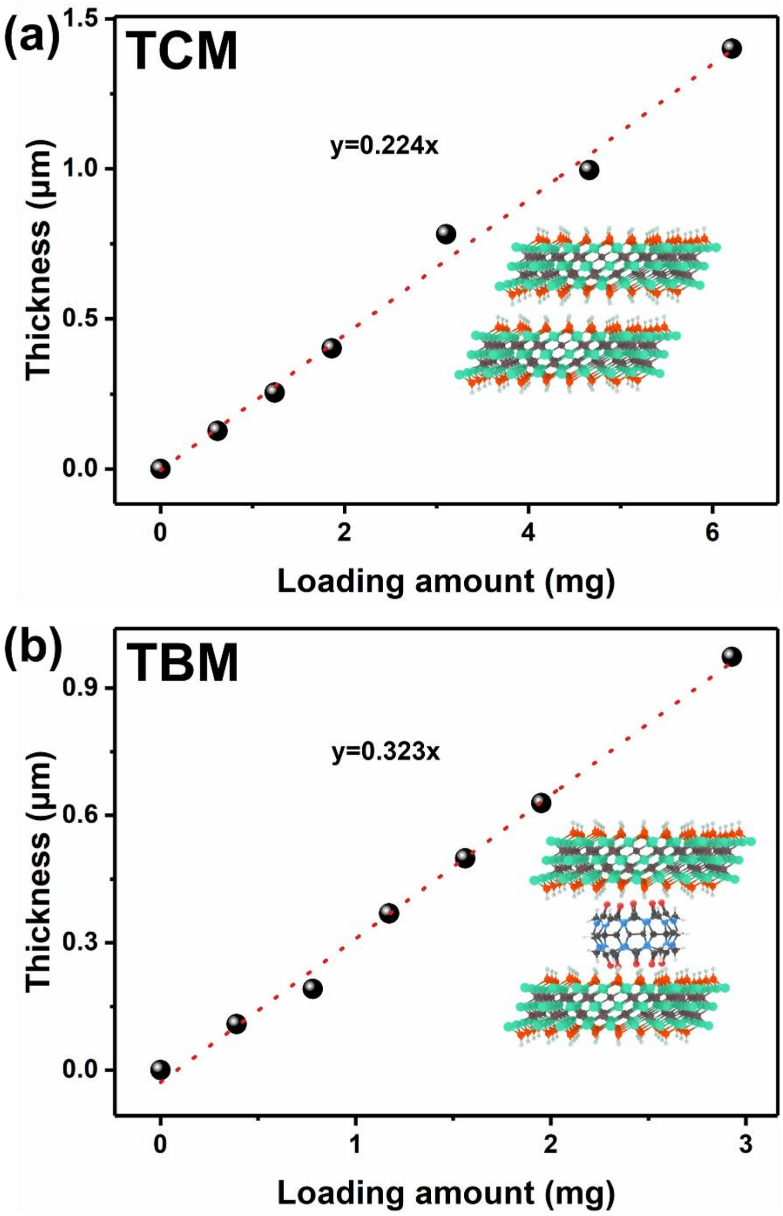

Figure S7. Variation of the membrane thickness as a function of MXene sheet loading.

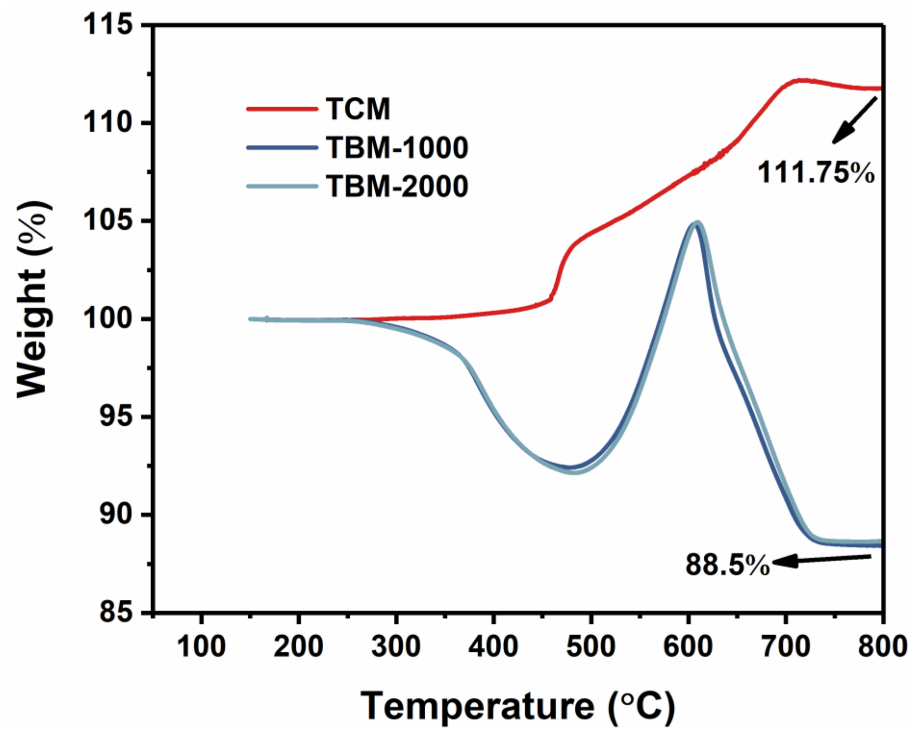

Figure S8. TGA curves of TCM, TBM-1000 and TBM-2000. The TGA curve of TCM shows that the mass of the final product (titanium dioxide) is 111.75% of the initial mass when MXene is heated to 800 °C in air. As the final product of TBM heated to 800 °C is also titanium dioxide, the content of MXene in TBM can be calculated based on the residual weight of the sample, that is, 88.5%/1.1175 = 79.2 wt%. The content of CB5 is thus 20.8 wt%. The thicknesses of TBM-1000 and TBM-2000 are 1000 nm and 2000 nm, respectively.

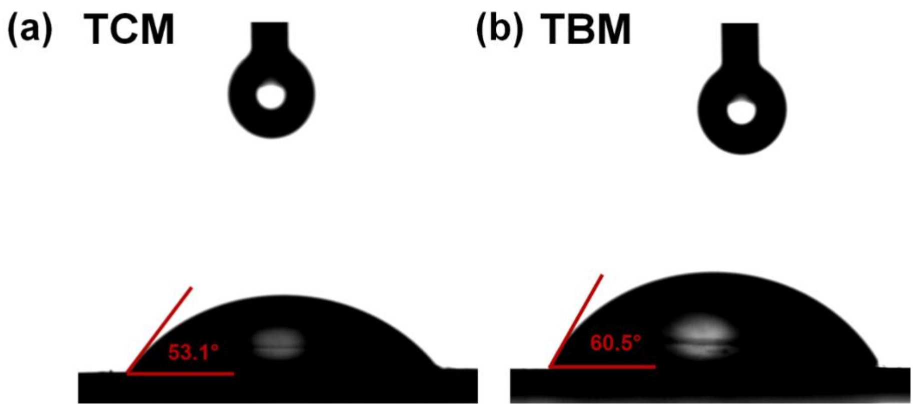

Figure S9. The water contact angle of (a)TCM and (b)TBM, both films are tested after vacuum drying to fully remove residual water from the surface.

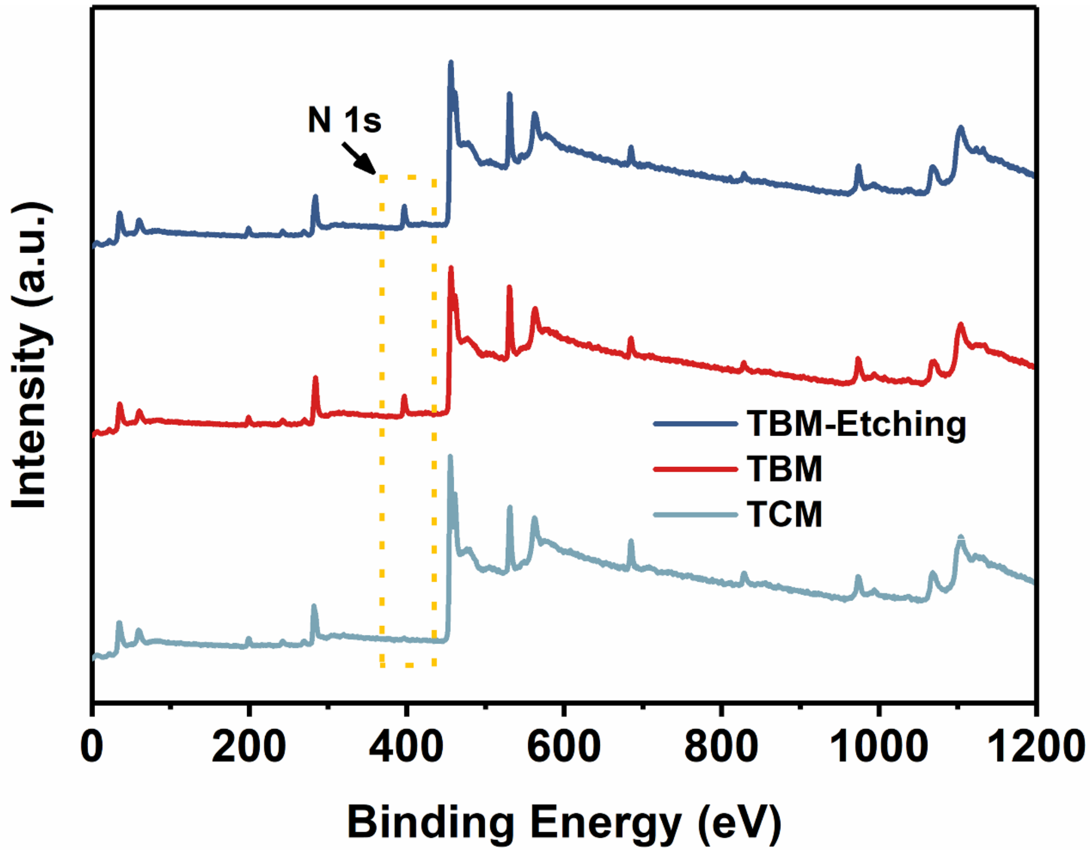

Figure S10. Survey scans of XPS spectra for TCM, TBM, and TBM after etching for 180 s.

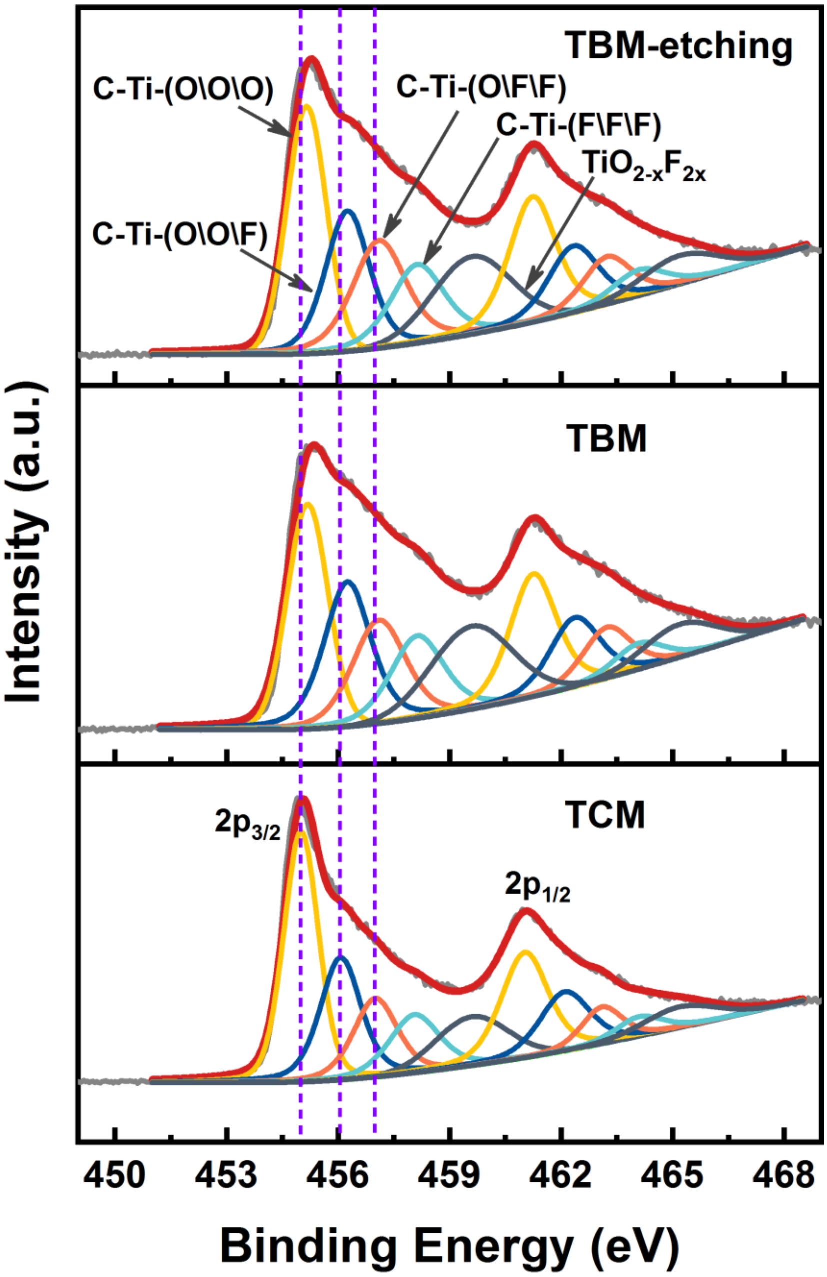

Figure S11. High-resolution XPS spectra fittings of TCM, TBM and TBM-Etching in Ti 2p region. Because each surface Ti in $Ti_3C_2T_x$ is surrounded by three atoms of O and/or F, and $TiO_{2-x}F_{2x}$ impurities may also be introduced in the synthesis process of MXene, the possible components are fitted as C–Ti–(O\O\O), C−Ti−(O\O\F), C−Ti−(O\F\F), C−Ti−(F\F\F) and $TiO_{2-x}F_{2x}$. The fitting results show that the binding energies of C–Ti–(O\O\O), C−Ti−(O\O\F), C−Ti−(O\F\F) bonds shift significantly to high energy after the immobilization of CB5, suggesting there is a strong interaction between CB5 and $Ti_3C_2T_x$ nanosheets, in which oxygen-containing groups may play an important role.

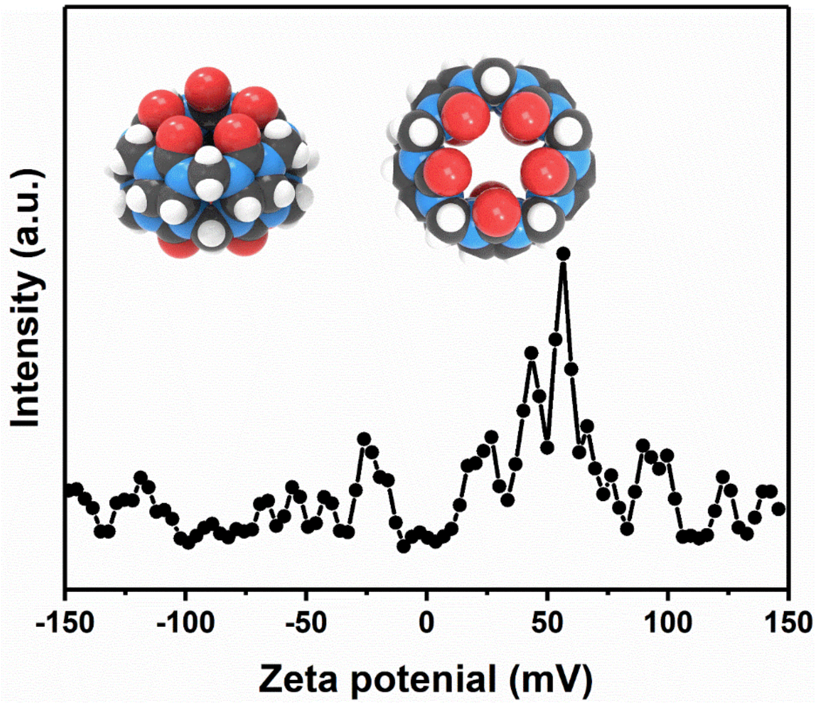

Figure S12. Zeta potential of CB5 in aqueous solution showing that CB5 is positively charged in solution.

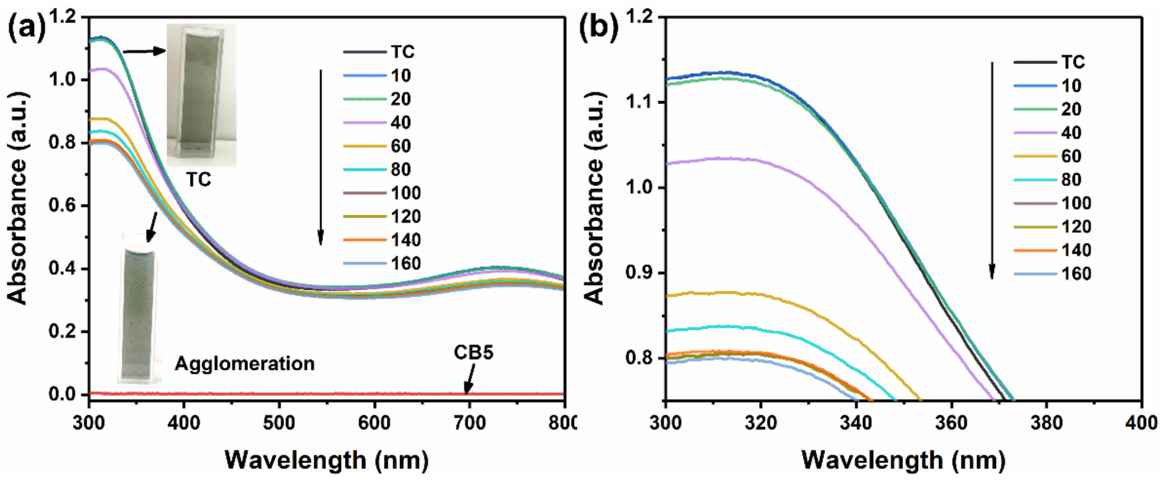

Figure S13. (a) UV-vis absorption spectra of TC during the CB5 titration process. (b) Enlarged view of the UV-vis absorption spectra in the 300-400 nm wavelength range.When the amount of CB5 solution added was less than 20 μL, the absorption spectrum of TC did not change significantly. As the addition of CB5 continued to increase, the absorbance dropped significantly, reflecting coagulation of MXene nanosheets. The initial concentration of nanosheets was 16 μg mL^{-1}.

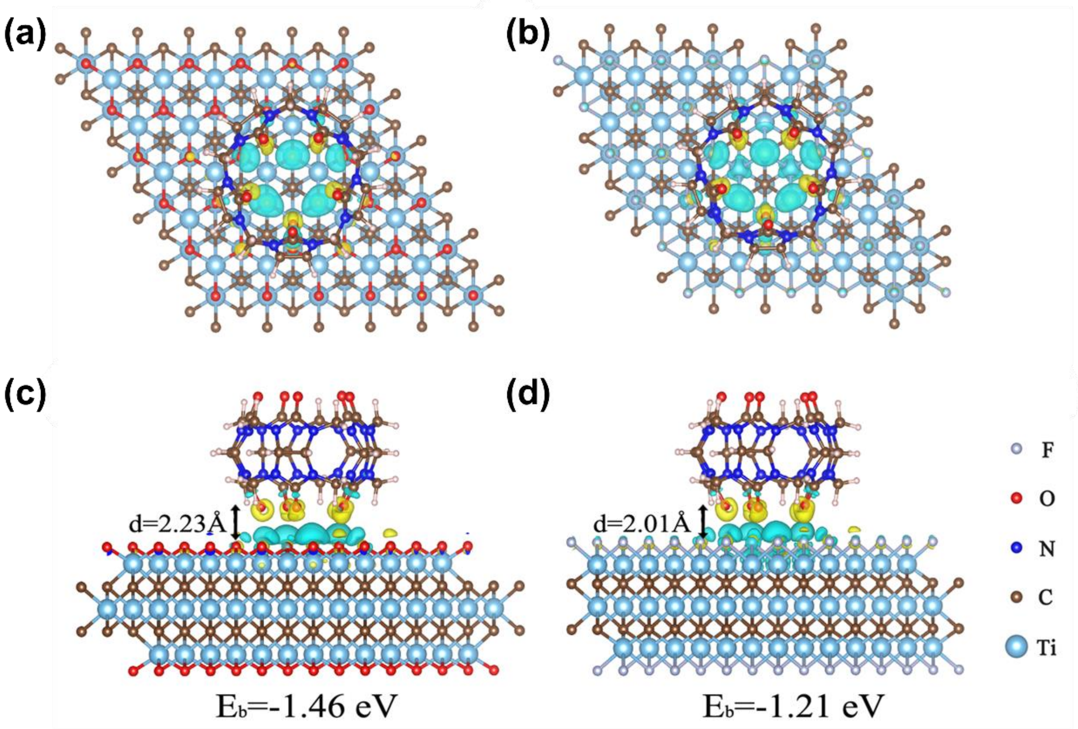

Figure S14. Charge density distribution of CB5-$Ti_3C_2T_x$ for T=O (a, c) and T=F (b, d). The isosurface level is set to 0.001 e bohr^{-3}. Yellow areas represents electron accumulation and blue area represents electron loss.

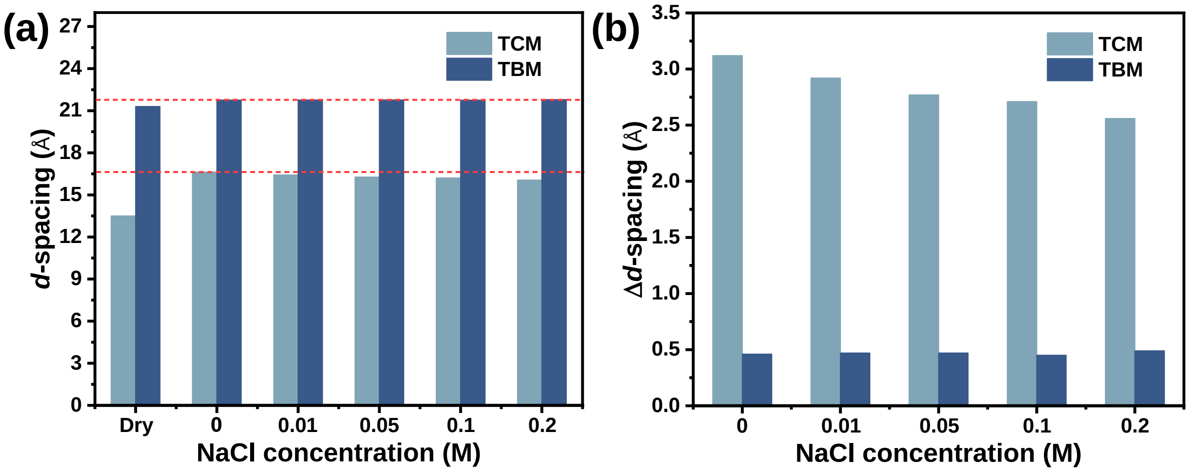

Figure S15. (a) D-spacing of TCM and TBM in different concentration gradients of NaCl (b) The corresponding enlarged d-spacing (Δd-spacing) of TCM and TBM compared to the dry sample.

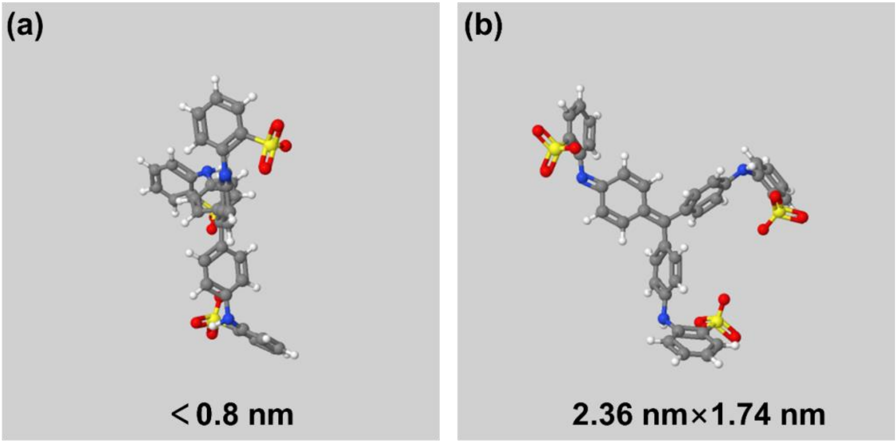

Figure S16. Schematic diagram of the optimized structure of methyl blue.

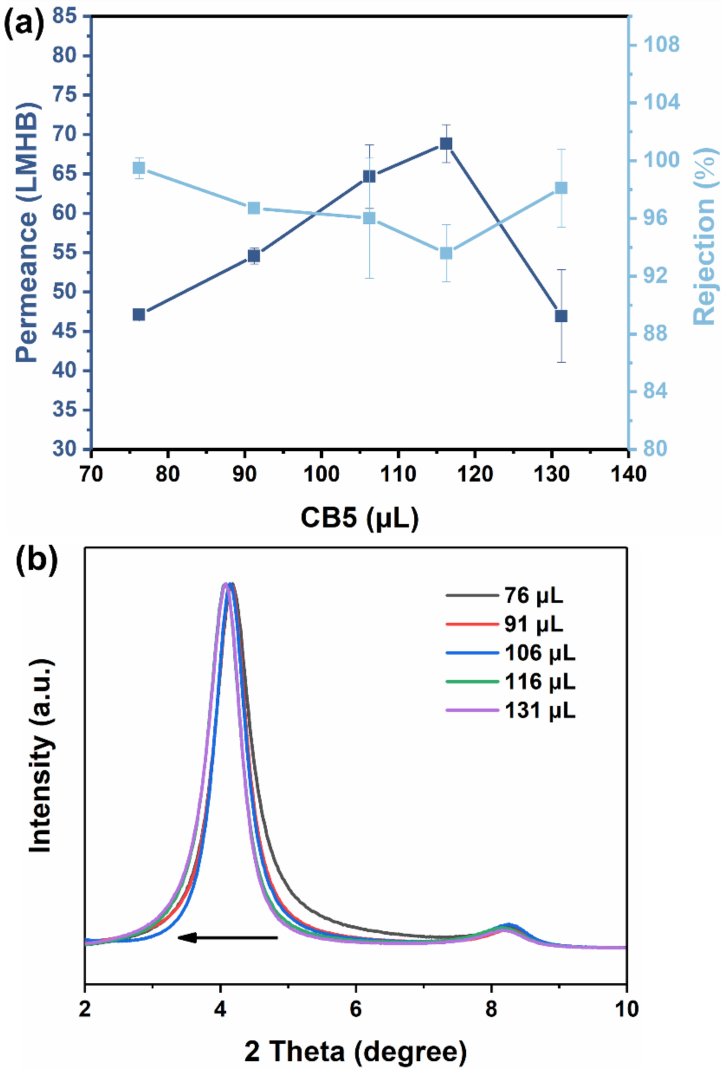

Figure S17. (a) Rejection rates of MB and the corresponding water permeance (LMHB = $Lm^{-2}h^{-1}bar^{-1}$) through the TBMs with different CB5 loadings. (b) The XRD patterns of corresponding TBMs after the nanofiltration tests.

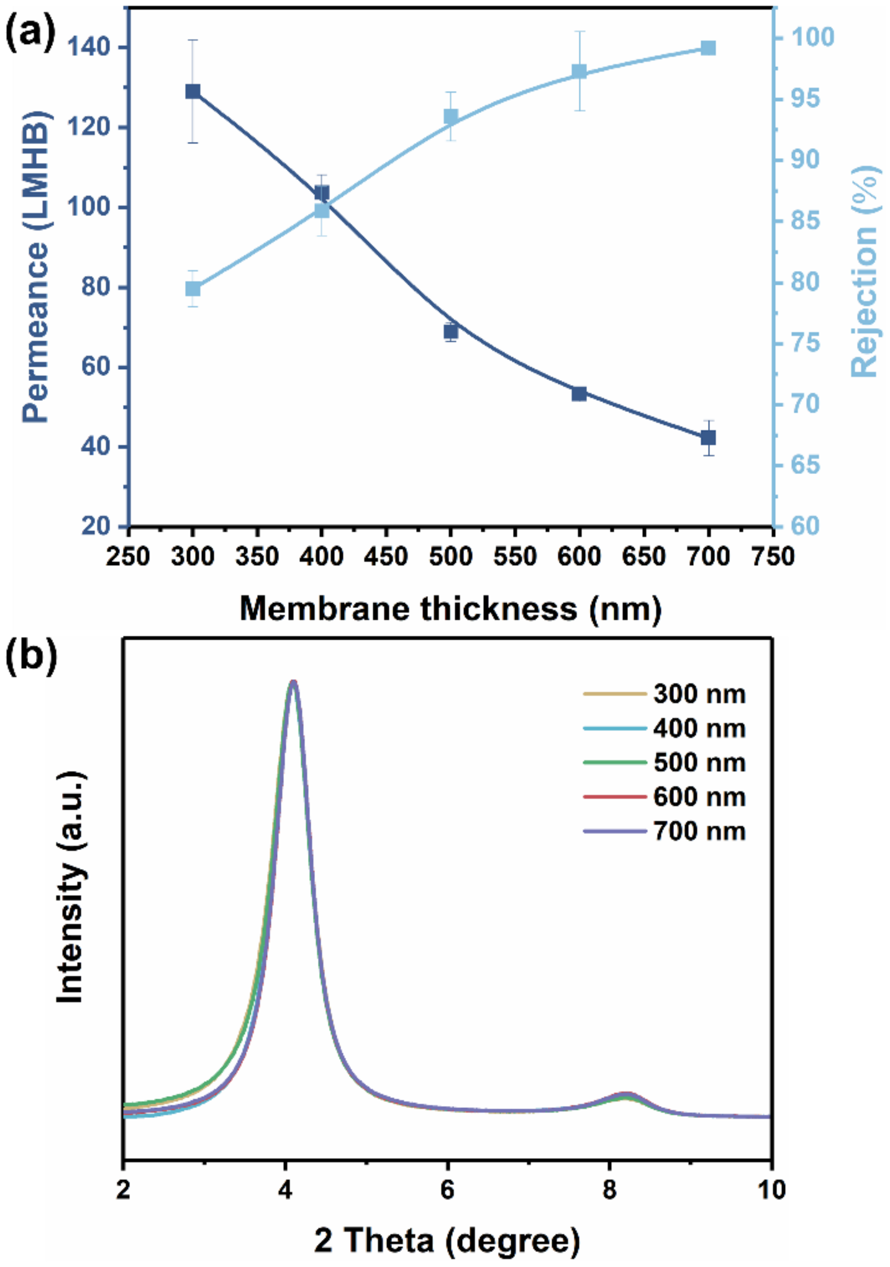

Figure S18. (a) Rejection rates of MB and the corresponding water permeance through the TBMs with different thicknesses. (b) The XRD patterns of the TBMs after the nanofiltration tests.

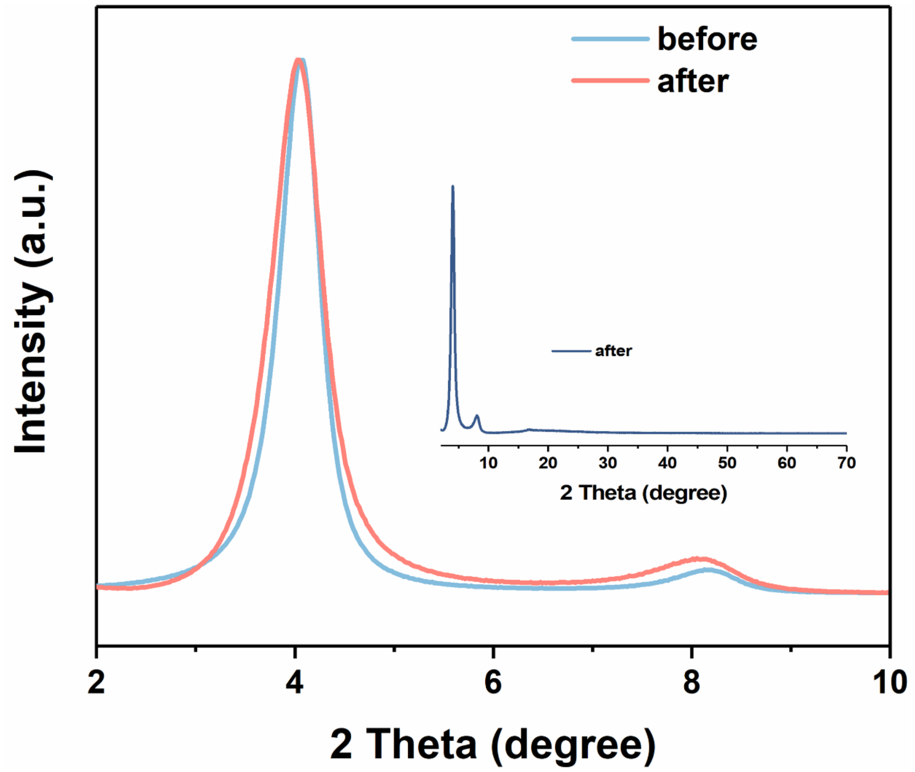

Figure S19. Comparison of XRD patterns before the test and after 30 cycles. The inserted pattern reflects that there is no impurity phase after the cycle test.

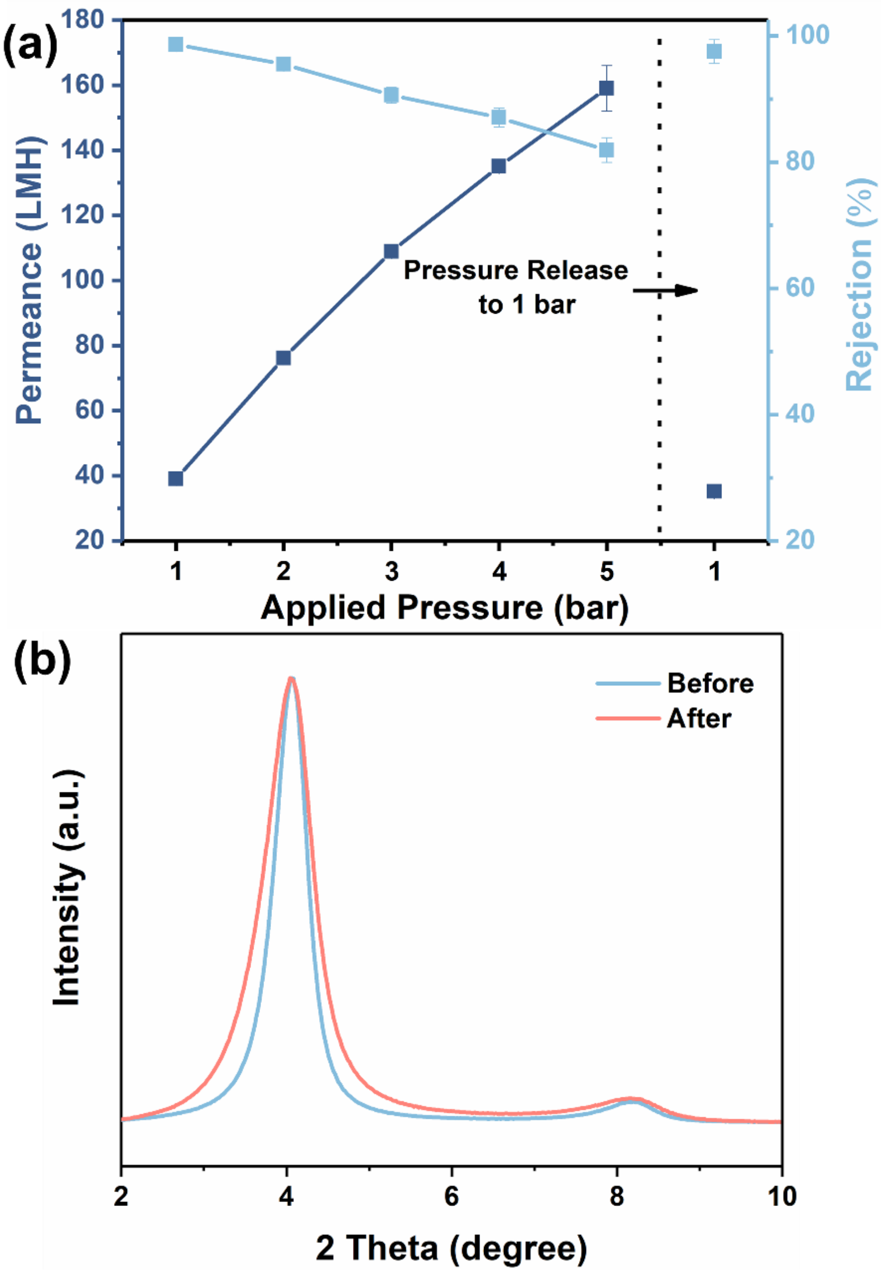

Figure S20. (a) Effect of pressure on the separation performance of TBM for MB. Data to the right of the dashed line was obtained after the pressure was released from 5 bar back down to 1 bar. (b) Comparison of XRD patterns before and after the test. The thickness of the membrane used here was 700 nm.

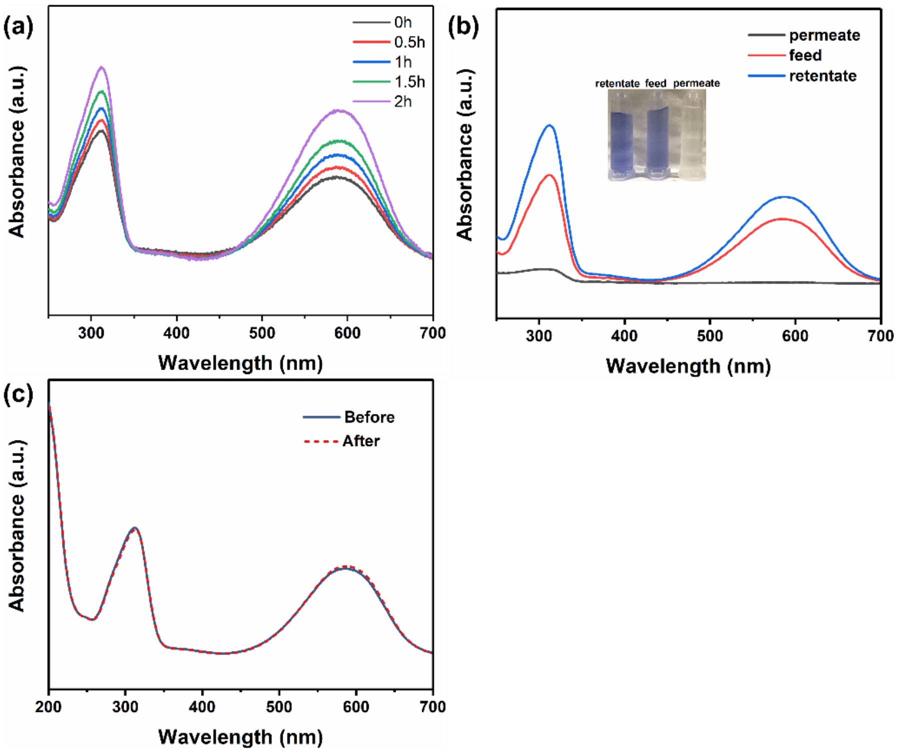

Figure S21. (a) UV/Vis spectra of MB aqueous solutions on the retentate side with time. (b) UV/Vis spectra of the permeate, the feed, and the retentate solutions. The total amount of molecules from both the permeate and retentate sides is very close to the original feed amount of the molecules, which implies that the MB are mostly rejected by the TBM rather than being absorbed or reacted with the membrane. (c) UV/Vis spectra of MB aqueous solutions before and after the adsorption using the TBM as an adsorbent for 4 hours.

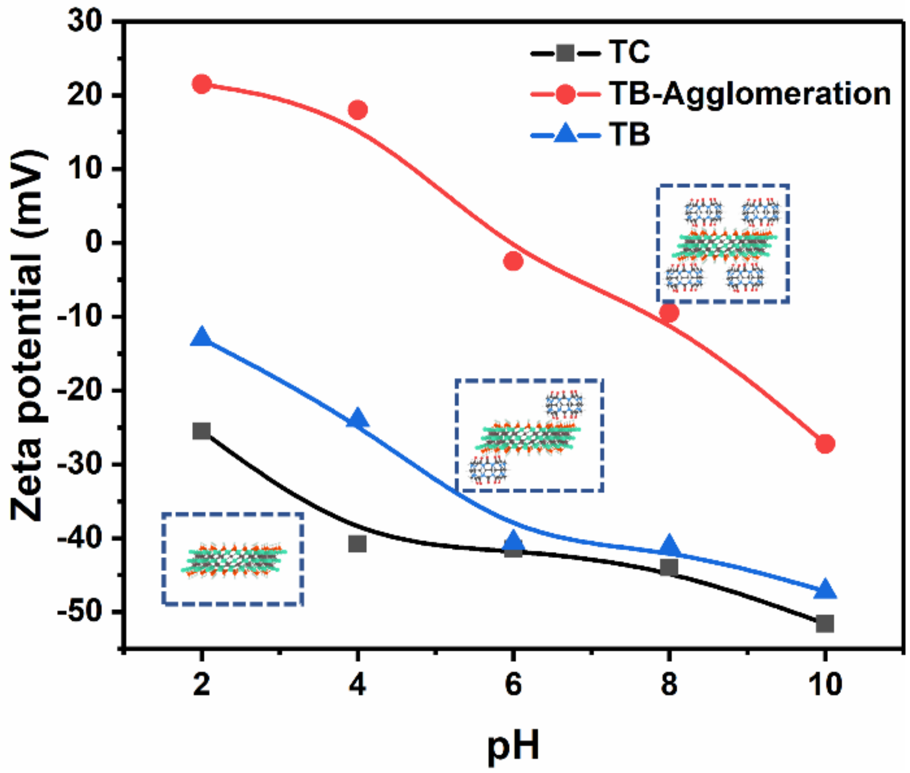

Figure S22. Zeta potential data of the nanosheets before and after the addition of CB5. With the addition of CB5 to the MXene surface, the surface potential increases. The surface charges of all three samples are negative under near-neutral pH conditions, although the value of MXene-CB5 agglomerates is close to zero due to the excessive introduction of CB5.

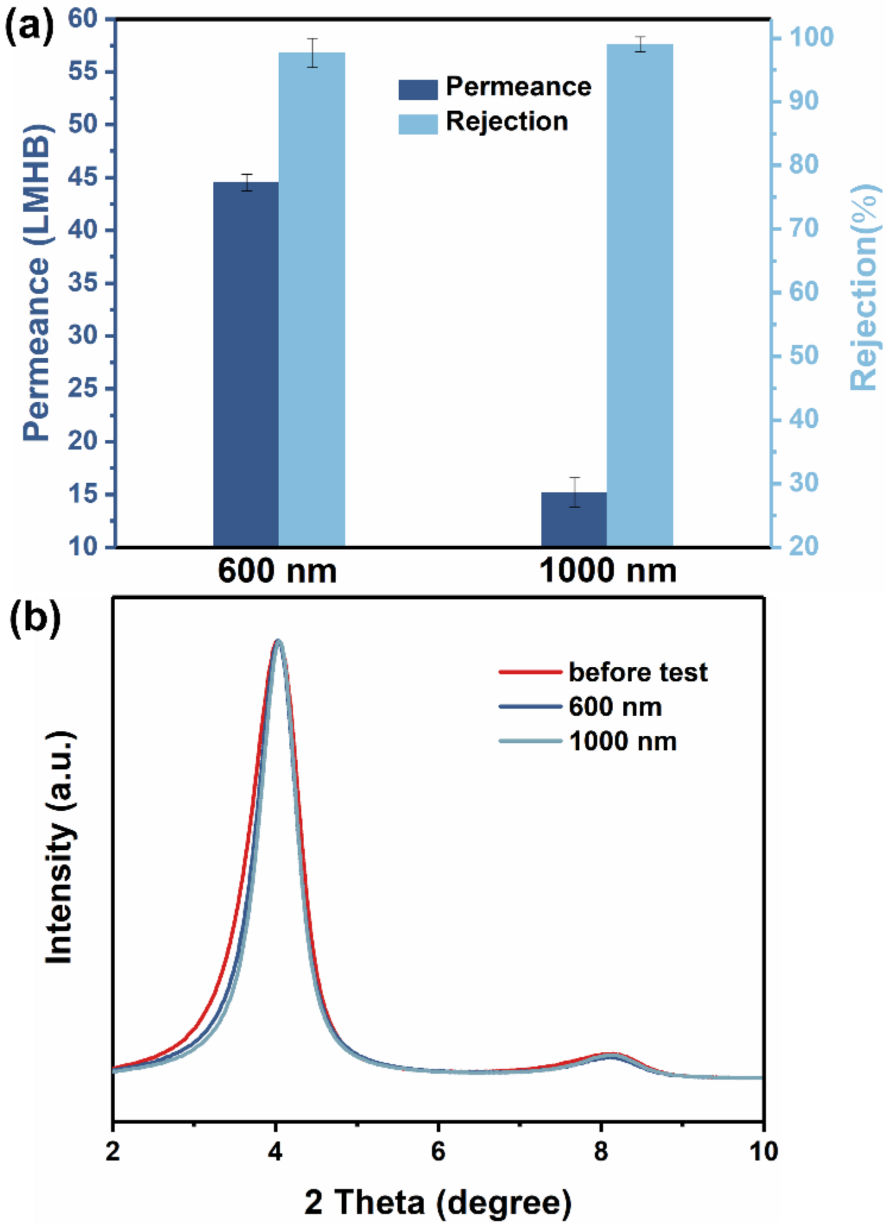

Figure S23. (a) Separation performance of TBM for U (10 mg L^{-1}, pH 9.0). (b) Corresponding XRD patterns of TBM before and after the test.

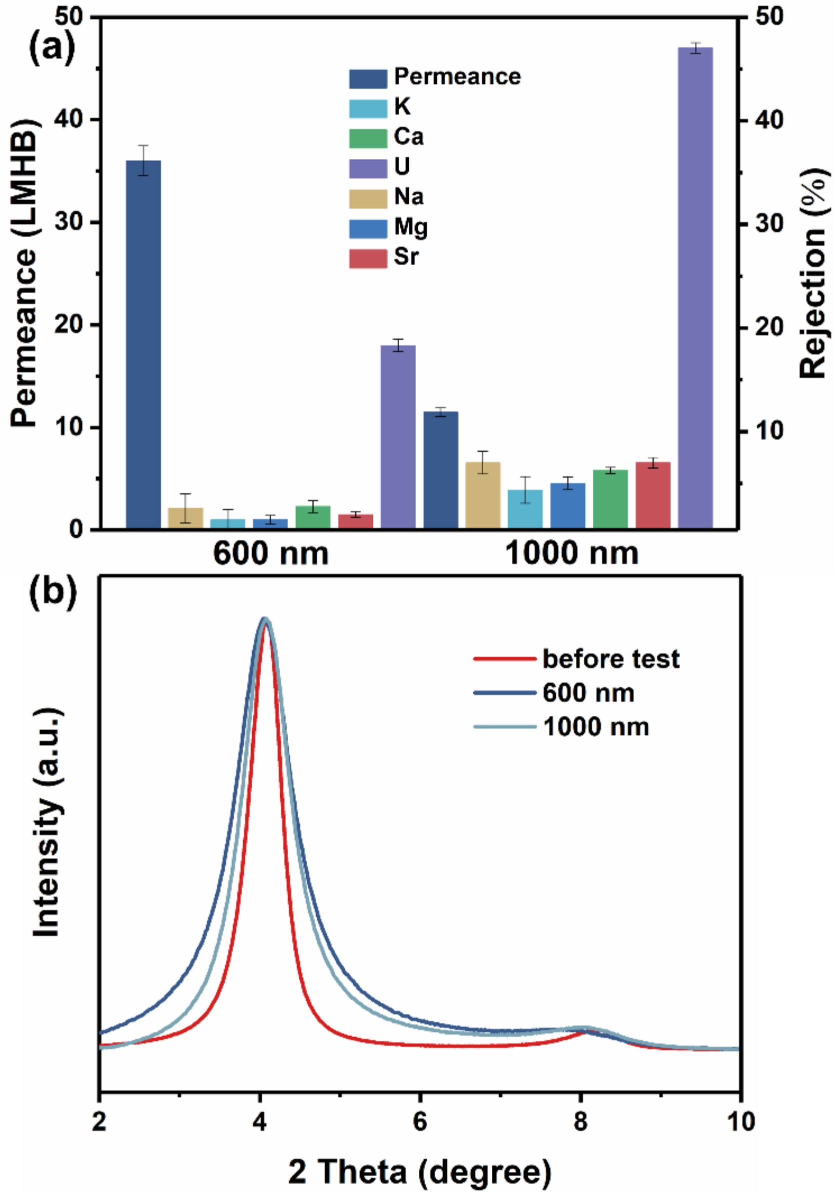

Figure S24. (a) Separation performance of TBM for uranyl carbonate in simulated seawater (10 mg L^{-1} uranium, pH 9.0). (b) Corresponding XRD patterns before and after the test. Ion concentrations: Na-11000 mg L^{-1}, K-390 mg L^{-1}, Mg-1300 mg L^{-1}, Ca-410 mg L^{-1}, Sr-13 mg L^{-1}.

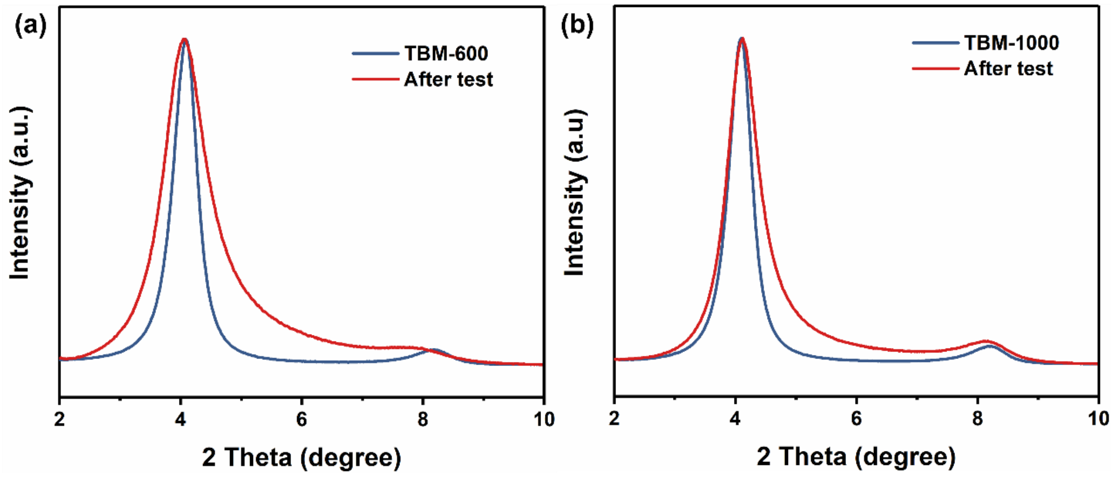

Figure S25. Changes in XRD patterns of TBM with membrane thicknesses (a) 600 nm and (b) 1000 nm, before and after natural seawater filtration testing. (a) 600 nm, (b) 1000 nm.

Table S1. Summary of XPS peak fittings of Ti 2p spectra of TCM, TBM and TBM-Etching.

Sample	Region	BE(eV)	FWHM(eV)	Fraction	Assigned to
TCM	Ti $2p_{3/2}(2p_{1/2})$	455.0(461.0)	1.1(1.5)	0.357	C−Ti−(O\O\O)
		456.1(462.1)	1.3(1.6)	0.224	C−Ti−(O\O\F)
		457.0(463.1)	1.4(1.3)	0.155	C−Ti−(O\F\F)
		458.1(464.1)	1.6(1.5)	0.131	C−Ti−(F\F\F)
		459.6(465.2)	2.3(2.3)	0.133	$TiO_{2-x}F_{2x}$
TBM	Ti $2p_{3/2}(2p_{1/2})$	455.2(461.3)	1.3(1.5)	0.262	C−Ti−(O\O\O)
		456.3(462.4)	1.5(1.6)	0.226	C−Ti−(O\O\F)
		457.1(463.2)	1.7(1.6)	0.176	C−Ti−(O\F\F)
		458.1(464.1)	1.7(1.6)	0.147	C−Ti−(F\F\F)
		459.6(465.2)	2.6(2.3)	0.189	$TiO_{2-x}F_{2x}$
TBM-Etching	Ti $2p_{3/2}(2p_{1/2})$	455.2(461.2)	1.3(1.5)	0.280	C−Ti−(O\O\O)
		456.3(462.3)	1.4(1.6)	0.203	C−Ti−(O\O\F)
		457.1(463.2)	1.7(1.6)	0.197	C−Ti−(O\F\F)
		458.1(464.1)	1.7(1.7)	0.144	C−Ti−(F\F\F)
		459.6(465.2)	2.5(2.4)	0.176	$TiO_{2-x}F_{2x}$

Table S2. Comparison of the separation performance of various membranes for methyl blue.

Membrane	Operation Pressure (bar)	Permeance ($L\ m^{-2}h^{-1}bar^{-1}$)	Rejection (%)	References
PEI/PAA/PVA/GA	5	0.85	87.3	[3]
ZIF-8/PSS	5	26.5	98.6	[4]
CMCNa/PP	0.8	8.25	99.75	[5]
PDDA/PSS	6	8.25	92	[6]
ZIF-12/PAN	2	27.2	99.4	[7]
ZIF-8/PSS	5	21	98.6	[8]
COF-LZU1	5	48.6	99.2	[9]
PDDA/GO	5	14.1	98.2	[10]
Mul-EGO-60	1	21.5	98	[11]
f-GOm	1	30.2	93.7	[12]
ZIF-8@f-GOm	1	49.8	99.8	[12]
ZIF-8/PEI	2	33	99.6	[13]
Fe(III)-phos-(PEI)	2	5	99.6	[14]
PEI/PAA-CSH	4	6.05	98.9	[15]
PS/SiO_2	4	5.2	99.5	[16]
PDDA/MoS_2	4	19.45	98.6	[17]
GO/MoS_2	2	10.2	97.4	[18]
PEI/MXene	4	20.9	98.84	[19]
TBM-500 nm	1	69	93.6	This work
TBM-600 nm	1	53.5	97.3	This work
TBM-700 nm	1	42.3	99.1	This work

References:

[1] An, S. W.; Mei, L.; Hu, K. Q.; Zhang, Z. H.; Xia, C. Q.; Chai, Z. F.; Shi, W. Q., *Inorg. Chem.* **2020,** *59*, 943.

[2] Day, A.; Arnold, A. P.; Blanch, R. J.; Snushall, B., *J. Org. Chem.* **2001,** *66*, 8094.

[3] Wang, N.; Ji, S.; Zhang, G.; Li, J.; Wang, L., *Chem. Eng. J.* **2012,** *213*, 318.

[4] Zhang, R.; Ji, S.; Wang, N.; Wang, L.; Zhang, G.; Li, J. R., *Angew. Chem. Int. Ed.* **2014,** *126*, 9933.

[5] Yu, S.; Chen, Z.; Cheng, Q.; Lü, Z.; Liu, M.; Gao, C., *Sep. Purif. Technol.* **2012,** *88*, 121.

[6] Tang, H.; Ji, S.; Gong, L.; Guo, H.; Zhang, G., *Polym. Chem.* **2013,** *4*, 5621.

[7] Wang, N.; Li, X.; Wang, L.; Zhang, L.; Zhang, G.; Ji, S., *ACS Appl. Mater. Interfaces* **2016,** *8*, 21979.

[8] Wang, N.; Liu, T.; Shen, H.; Ji, S.; Li, J.-R.; Zhang, R., *AlChE J.* **2016,** *62*, 538.

[9] Fan, H.; Gu, J.; Meng, H.; Knebel, A.; Caro, J., *Angew. Chem. Int. Ed.* **2018,** *57*, 4083.

[10] Wang, L.; Wang, N.; Li, J.; Li, J.; Bian, W.; Ji, S., *Sep. Purif. Technol.* **2016,** *160*, 123.

[11] Wang, Q.; Zhao, G.; Li, C.; Meng, H., *J. Membr. Sci.* **2019,** *586*, 177.

[12] Zhang, W. H.; Yin, M. J.; Zhao, Q.; Jin, C. G.; Wang, N.; Ji, S.; Ritt, C. L.; Elimelech, M.; An, Q. F., *Nat. Nanotechnol.* **2021,** *16*, 337.

[13] Yang, L.; Wang, Z.; Zhang, J., *J. Membr. Sci.* **2017,** *532*, 76.

[14] Li, P.; Wang, Z.; Yang, L.; Zhao, S.; Song, P.; Khan, B., *J. Membr. Sci.* **2018,** *555*, 56.

[15] Guo, H.; Ma, Y.; Qin, Z.; Gu, Z.; Cui, S.; Zhang, G., *ACS Appl. Mater. Interfaces* **2016,** *8*, 23379.

[16] Ding, W.; Zhuo, H.; Bao, M.; Li, Y.; Lu, J., *Chem. Eng. J.* **2017,** *330*, 337.

[17] Zhou, J.; Qin, Z.; Lu, Y.; Li, X.; An, Q.; Ji, S.; Wang, N.; Guo, H., *Journal of the Taiwan Institute of Chemical Engineers* **2018,** *84*, 196.

[18] Zhang, P.; Gong, J.-L.; Zeng, G.-M.; Song, B.; Cao, W.; Liu, H.-Y.; Huan, S.-Y.; Peng, P., *J. Membr. Sci.* **2019,** *574*, 112.

[19] Li, J.; Li, L.; Xu, Y.; Zhu, J.; Liu, F.; Shen, J.; Wang, Z.; Lin, J., *Chem. Eng. J.* **2022,** *427*.
